# Mindin regulates fibroblast subpopulations through distinct Src family kinases during fibrogenesis

**DOI:** 10.1172/jci.insight.173071

**Published:** 2024-12-31

**Authors:** Sunny Kataria, Isha Rana, Krithika Badarinath, Rania F. Zaarour, Gaurav Kansagara, Sultan Ahmed, Abrar Rizvi, Dyuti Saha, Binita Dam, Abhik Dutta, Ravindra K. Zirmire, Edries Yousaf Hajam, Pankaj Kumar, Akash Gulyani, Colin Jamora

**Affiliations:** 1 IFOM-inStem Joint Research Laboratory, Centre for Inflammation and Tissue Homeostasis, Institute for Stem Cell Science and Regenerative Medicine (inStem), Bangalore, Karnataka, India.; 2 Department of Life Sciences, Shiv Nadar Institution of Eminence, Gautam Buddha Nagar, India.; 3 National Centre for Biological Sciences, Gandhi Krishi Vigyan Kendra Post, Bangalore, Karnataka, India.; 4 Shanmugha Arts, Science, Technology and Research Academy (SASTRA) University, Thanjavur, Tamil Nadu, India.; 5 Manipal Academy of Higher Education, Manipal, India.; 6 Integrative Chemical Biology, inStem, Bangalore, Karnataka, India.

**Keywords:** Dermatology, Inflammation, Autoimmune diseases, Fibrosis, Signal transduction

## Abstract

Fibrosis results from excessive extracellular matrix (ECM) deposition, which causes tissue stiffening and organ dysfunction. Activated fibroblasts, central to fibrosis, exhibit increased migration, proliferation, contraction, and ECM production. However, it remains unclear if the same fibroblast performs all of the processes that fall under the umbrella term of “activation.” Owing to fibroblast heterogeneity in connective tissues, subpopulations with specific functions may operate under distinct regulatory controls. Using a transgenic mouse model of skin fibrosis, we found that Mindin (also known as spondin-2), secreted by *Snail-*transgenic keratinocytes, differentially regulates fibroblast subpopulations. Mindin promotes migration and inflammatory gene expression in SCA1+ dermal fibroblasts via Fyn kinase. In contrast, it enhances contractility and collagen production in papillary CD26^+^ fibroblasts through c-Src signaling. Moreover, in the context of the fibrotic microenvironment of the tumor stroma, we found that differential responses of resident fibroblast subpopulations to Mindin extend to the generation of functionally heterogeneous cancer-associated fibroblasts. This study identifies Mindin as a key orchestrator of dermal fibroblast heterogeneity, reshaping cellular dynamics and signaling diversity in the complex landscapes of skin fibrosis and cancer.

## Abstract 2

The matricellular protein Mindin elicits different responses from specific dermal fibroblasts subpopulations which together drives fibrogenesis

## Introduction

Fibrosis is a leading cause of death in many chronic diseases, and the central players in this pathophysiology are fibroblasts. The key events in its pathogenesis can be conceptualized as a chronic exaggeration of wound-healing processes that lead to scarring (1–3). The processes involved in wound healing can be broadly divided into four overlapping phases — hemostasis, inflammation, proliferation, and remodeling (4). During the inflammatory phase, the fibroblasts are activated and migrate to the site of injury. The fibroblasts at the wound site proliferate and differentiate into myofibroblasts, where they perform several functions and coordinate multiple aspects of the wound-healing program (5). While the number of activated fibroblasts dissipates following normal wound healing, this is not the case with fibrotic diseases, which are associated with the persistent presence of activated fibroblasts (5, 6). However, to understand what causes the persistence of activated fibroblasts in pathological scenarios such as fibrosis, it is important to understand what activates them and how they remain in a chronically active state.

A major complication in understanding fibroblast regulation and function is that fibroblasts in the dermal compartment of the skin are spatially and functionally heterogeneous (7–9). Fibroblasts in the neonatal skin arise from two distinct lineages. The upper lineage, marked by CD26, forms the papillary dermis and contributes to hair follicle generation, arrector pili muscle, and epidermal homeostasis (8, 9). The lower dermal lineage, marked by SCA1, contributes to the deposition of the majority of fibrous collagen and can differentiate into adipocytes to maintain the dermal white adipose layer (8–10). In wound healing, the SCA1^+^ fibroblasts are the first cells recruited to the wound bed where they repopulate the extracellular matrix (ECM), while recruitment of papillary fibroblasts is associated with reepithelialization and hair follicle generation (9).

These subpopulations also display heterogeneous responses in fibrosis (10–13). Nonetheless, the contributions of various fibroblast subpopulations in response to profibrotic stimuli and the molecular mechanisms underlying their differential activities remain largely unknown. To begin uncovering these molecular mechanisms, we utilized a *Snail*-transgenic (*Snail*-Tg) mouse model of skin fibrosis that mimics the overexpression of this transcription factor found in the epidermis of patients with scleroderma (SSc) (14, 15). Ectopic expression of snail is sufficient to induce phenotypes that recapitulate many diagnostic features of systemic SSc, including dermal thickening and fibrosis (15). It is also interesting to note that patients SSc also have a higher incidence of developing cancers (16), suggesting that insights into this fibrotic disease would likewise shed light on the factors driving tumorigenesis. In line with this, the *Snail*-Tg mice have also been shown to prime the skin toward the development of cutaneous squamous cell carcinoma (17, 18).

The mesenchymal compartment of fibrotic tissue has remarkable similarities to the stroma surrounding solid tumors (19, 20). The fibrosis associated with tumor stroma is driven by cancer-associated fibroblasts (CAFs) (21). Much like their counterparts in normal tissue, CAFs are heterogeneous in nature (20). One subpopulation of CAFs, known as myofibroblastic CAFs (myCAFs), expresses higher levels of α–smooth muscle actin (α-SMA), collagens, and other genes associated with myofibroblast functions (22). Another major subpopulation is the inflammatory CAFs (iCAFs), which majorly express inflammatory cytokines (22). Collectively, these different CAFs promote tumor-associated inflammation, regulate ECM remodeling of stroma, regulate cancer cell metabolism, promote survival and maintenance of cancer stem cells, and aid metastasis and chemoresistance (19, 20). Despite their important contributions to tumorigenesis, the origins of these different CAFs remain unknown. We have previously shown that *Snail*-Tg keratinocytes secrete Mindin (also known as Spondin-2; a member of the F-Spondin family) (18). Mindin has been implicated in response to injury (23, 24) and is overexpressed in multiple inflammatory and fibrotic diseases (25–30). It has also emerged as a prognostic and diagnostic biomarker for various carcinomas (31–35), and we have previously shown that Mindin is essential for tumorigenesis and fibrogenesis in the *Snail*-Tg mouse model (15, 18). This presented us with a unique opportunity to further investigate the role of Mindin in elucidating the regulation of the functional heterogeneity of fibroblasts in both tissue fibrosis and the tumor stroma.

## Results

### Snail-Tg skin has perturbed proportions and localization of fibroblast subpopulations.

**Figure 1:**
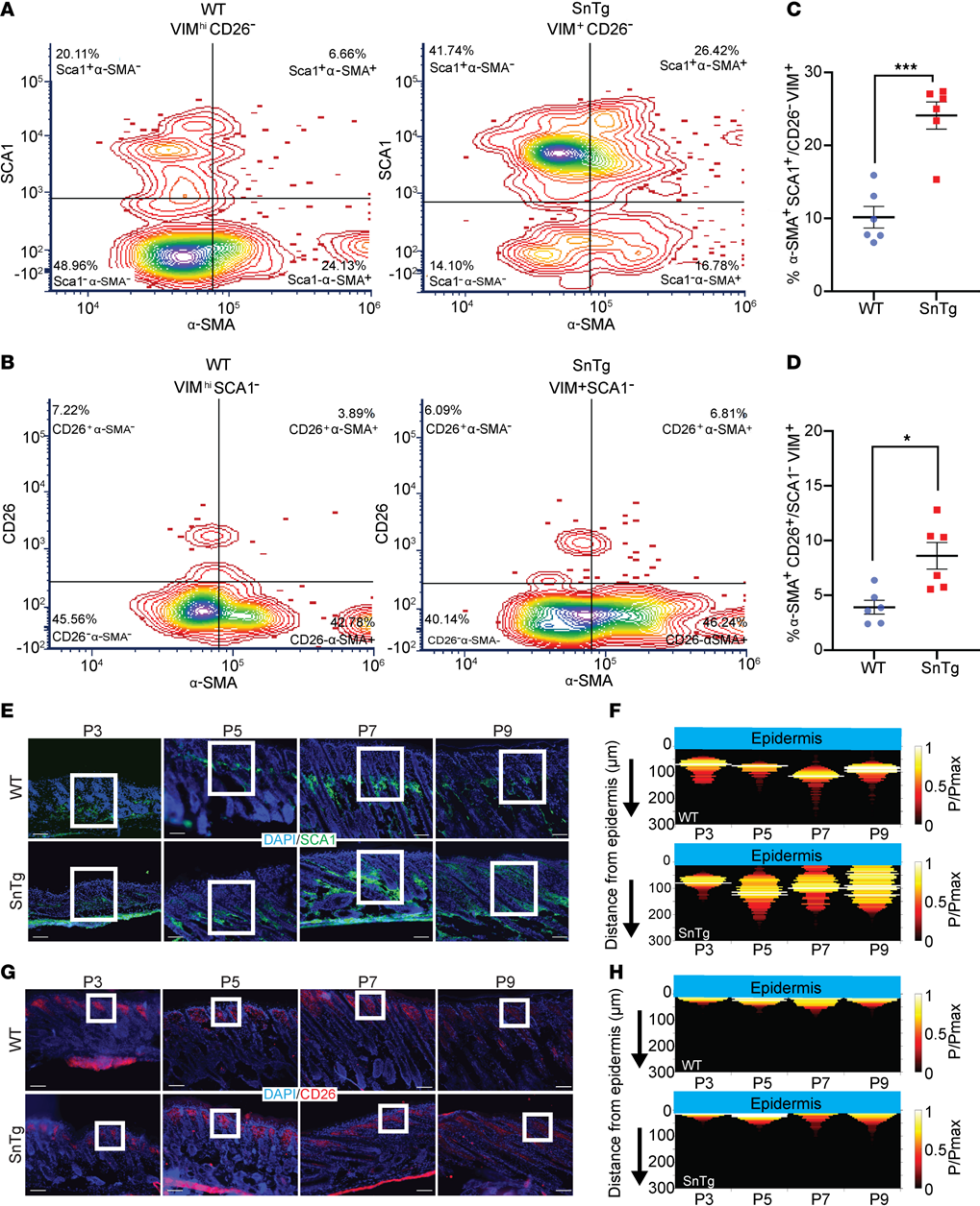
SCA1^+^ fibroblast localization is perturbed in the dermis of *Snail*-transgenic mice. Representative contour plot showing quadrants for (**A**) α-SMA^+^SCA1^+^/CD26^–^VIM^hi^, α-SMA^+^SCA1^-^/CD26^–^VIM^hi^, α-SMA^-^SCA1^+^/CD26^–^VIM^hi^ and α-SMA^–^SCA1^–^/CD26^–^VIM^hi^ and (**B**) α-SMA^+^CD26^+^/SCA1^–^VIM^hi^, α-SMA^+^CD26^–^/SCA1^–^VIM^hi^, α-SMA^–^CD26^+^/SCA1^–^VIM^hi^ and α-SMA^–^CD26^–^/SCA1^–^VIM^hi^ cells from P9 WT (left) and *Snail*-transgenic (*SnTg*) (right) mice. Individual value plots (mean ± SEM) of (**C**) the percentage of α-SMA^+^SCA1^+^/CD26^–^VIM^hi^ and (**D**) the percentage α-SMA^+^CD26^+^/SCA1^–^VIM^hi^ cells (*n* = 6; *P* values were calculated by Welch’s *t* test; *P < 0.05, ***P < 0.001). (**E**) SCA1^+^ fibroblasts (green) and nuclear staining with DAPI (blue) in WT and *SnTg* skin sections in P3, P5, P7, and P9 pups. The white boxes mark the insets shown in Supplemental Figure 1J. Note that the green stain at the bottom of the skin section is the autofluorescence of the paper used to keep the tissue uncurled during the embedding process. (**F**) Heatmap showing the probability of SCA1^+^ cells at a given distance below the epidermis in WT (top) and *SnTg* (bottom) mice. P3 (*n* =3 WT and *Snail Tg*), P5 (*n* = 2 WT and *n* = 4 *Snail Tg*), P7 (*n* = 3 WT and *n* = 4 *Snail Tg*), and P9 (*n* = 6 WT and *n* = 8 *Snail Tg*). (**G**) CD26^+^ fibroblasts (red) and nuclear staining with DAPI (blue) in WT and *SnTg* skin sections from P3, P5, P7, and P9 pups. The white boxes mark the insets shown in Supplemental Figure 1L as magnified areas. The boxed areas are shown at higher magnification in Supplemental Figure 1L. Note that the red stain at the bottom of the skin section is the autofluorescence of the paper used to keep the tissue uncurled during the embedding process. (**H**) Heatmap showing the probability of CD26^+^ cells at a given distance below the epidermis in WT (top) and *SnTg* (bottom) at P3 (*n* = 2 WT and *n* = 3 *Snail Tg*), P5 (*n* = 2 WT and *n* = 3 *Snail Tg*), P7 (*n* = 3 WT and *n* = 3 *Snail Tg*), and P9 (*n* = 4 WT and *n* = 6 *Snail Tg*).

We have previously shown that α-SMA, a marker for activated fibroblasts, is upregulated in the *Snail*-Tg dermis as early as P9 (36). The dermal fibroblasts can be spatially subdivided into two major subpopulations, papillary fibroblasts in the upper dermis and reticular and hypodermal fibroblasts in the lower dermis (37, 38). These subpopulations can be isolated based on surface markers, CD26 for papillary and SCA1 for lower reticular/hypodermal fibroblasts (8–10). The parameters of fibroblast heterogeneity, such as the proportion of specific fibroblast subtypes, may be altered during repair and disease conditions (39). To determine the proportions of fibroblast subpopulations, we performed flow cytometry using high vimentin (VIM) expression as a classical marker for fibroblasts (40, 41). We observed a significant increase in the proportion of VIM^hi^ cells that expressed SCA1 (lower dermal fibroblasts), while the proportion of VIM^hi^ cells that express CD26 but not SCA1 (papillary fibroblasts) showed a small decrease of approximately 1% in the *Snail*-Tg skin (Supplemental Figure 1, A–E; supplemental material available online with this article; https://doi.org/10.1172/jci.insight.173071DS1). In addition, there was no significant change in the VIM^hi^ cells expressing both CD26 and SCA1 in the *Snail*-Tg skin (Supplemental Figure 1F). However, the pool of VIM^hi^ fibroblasts that did not express SCA1 and CD26 (SCA1^–^CD26^–^) was decreased (Supplemental Figure 1G), and this suggests that this pool can possibly be the source for the increase in lower dermal fibroblasts (SCA1^+^CD26^–^/VIM^hi^) in the *Snail*-Tg skin. Moreover, we observed that nonfibroblasts (VIM^–^) expressing either SCA1 or CD26 were unchanged in the *Snail-*Tg background (Supplemental Figure 1, H and I). We probed for the expression of α-SMA in CD26^+^/SCA1^–^/VIM^hi^ and SCA1^+^/CD26^–^/VIM^hi^ cells to determine the activation status of these two subpopulations of fibroblasts. Both subpopulations showed an increase in the number of activated cells in the *Snail*-Tg background relative to their WT controls (Figure 1, A–D, and Supplemental Table 1). Altogether, this demonstrates that in the *Snail*-Tg background the proportions of fibroblast subpopulations are perturbed and both subpopulations contribute to the pool of activated cells.

Interestingly, when we investigated the spatial organization of the cells in vivo, we found that SCA1^+^ fibroblasts were progressively recruited from the lower dermis to the epidermal-dermal junction (Figure 1, E and F, and Supplemental Figure 1, J and K). Analysis of the probability distribution of SCA1^+^ fibroblasts as a function of distance from the epidermis revealed that SCA1^+^ cells were distributed between 50 and 150 μm away from the epidermis in WT skin at all postnatal ages analyzed (Figure 1F and Supplemental Figure 1K). In *Snail*-Tg skin, the SCA1^+^ cells were first noticed to have a differential localization at P5, and the probability of finding these cells near the epidermis gradually increased over time. Interestingly, the localization of CD26^+^ fibroblasts remained largely invariant between the WT and *Snail*-Tg skin (Figure 1, G and H, and Supplemental Figure 1, L and M).

### Mindin induces migration of SCA1^+^ fibroblasts.

**Figure 2:**
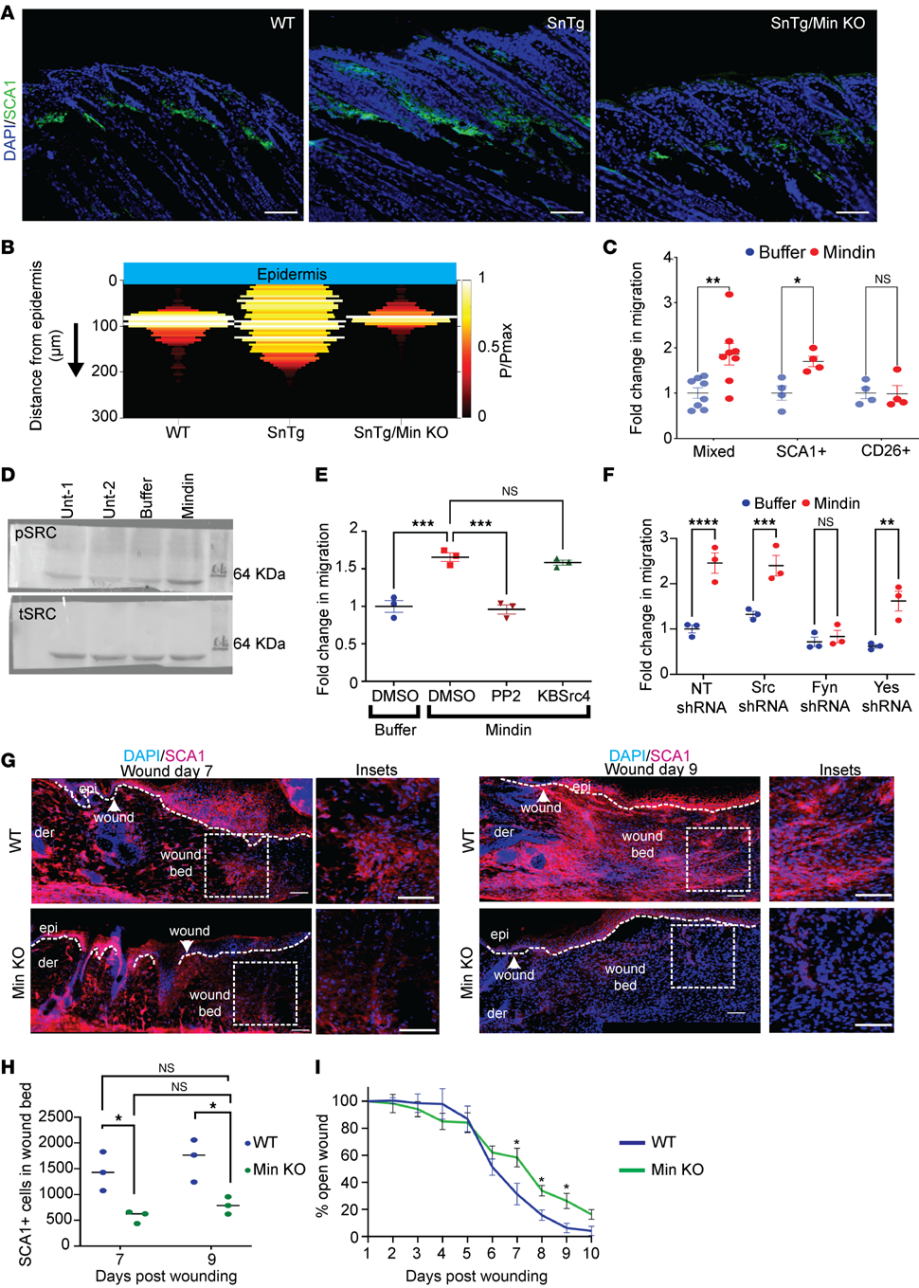
Mindin induces migration of SCA1^+^ fibroblasts via Fyn kinase. (**A**) IF staining for SCA1 in P9 WT, *SnTg*, and *SnTg/Mindin-*KO (*SnTg/Min*-KO) skin (scale bar: 50 μm). (**B**) Heatmap showing the probability of SCA1^+^ cells at a given distance below the epidermis in WT (*n* = 6), *SnTg* (*n* = 8), and *SnTg/Min*-KO (*n* = 4) skin. Data for WT and *SnTg* are the same as in Figure 1F. (**C**) Transwell assay to measure migration of mixed, SCA1^+^, and CD26^+^ fibroblasts with either buffer or Mindin as a potential chemoattractant (*n* ≥ 4). (**D**) Amount of phosphorylated SRC (pSRC) and total SRC (tSRC) proteins in fibroblasts treated with either buffer or Mindin for 15 minutes. (**E**) Transwell assay with SCA1^+^ fibroblasts stimulated with buffer or Mindin in the presence of DMSO, PP2 (10 μM), or KbSrc4 (10 μM) (*n* = 3). (**F**) Transwell assay with SCA1^+^ fibroblasts transduced with nontargeting (NT), *Src*, *Fyn*, or *Yes* shRNA with buffer or Mindin as a chemoattractant (*n* = 3). (**G**) IF for SCA1 in WT and *Min*-KO day 7 and day 9 skin wounds. (The images were stitched using FIJI ImageJ stitching tool, ref. 84; scale bar: 50 μm.) White boxes denote regions shown at higher magnification on the right-hand side of each image. (**H**) Quantification of SCA1^+^ cells in the wound beds day 7 and day 9 after wounding of WT and *Min*-KO mice (*n* = 3 mice) (**I**) Percentage wound closure in WT and *Min*-KO mice with regard to wound size on day 1 (*n* = 3 mice, 2 wounds per mice). Data represent the mean ± SEM. *P* values were calculated by Welch’s *t* test (**C** and **I**), 1-way ANOVA followed by Tukey’s post hoc analysis (**E**), and 2-way ANOVA followed by post hoc Šídák’s multiple comparisons test (**F** and **H**). *P < 0.05, **P < 0.01, ***P < 0.001, ****P < 0.0001; NS, *P* > 0.05.

We recently observed that the matricellular protein Mindin is secreted from *Snail*-Tg keratinocytes (15, 18) and is required for the expression of inflammatory cytokines in dermal fibroblasts (15). Mindin was also found to be necessary for dermal thickening, as measured by H&E staining, and for fibrosis, as indicated by collagen I levels in the transgenic mouse (15). In addition, we found that Mindin deficiency reduced the number of activated dermal fibroblasts marked by α-SMA in *Snail*-Tg*/Mindin*-knockout (*Min*-KO) skin (Supplemental Figure 2, A and B). To test whether Mindin is also required for the relocalization of SCA1^+^ fibroblasts toward the epidermal-dermal junction in the *Snail*-Tg skin, we stained SCA1^+^ fibroblasts (Figure 2A) and quantified their localization in P9 WT, *Snail*-Tg, and *Snail*-Tg*/Min*-KO skin (Figure 2B and Supplemental Figure 2C). The localization of SCA1^+^ cells in the *Snail*-Tg*/Min*-KO skin was similar to that in WT skin, indicating that Mindin is required for the relocalization of the cells from the lower dermis toward the epidermal-dermal junction. Furthermore, conditioned media from a Mindin-expressing CHO cell line induced migration of fibroblasts in vitro whereas the control CHO-conditioned media had no effect (Supplemental Figure 2, D and E). To test whether Mindin is sufficient to change SCA1^+^ fibroblast localization, we assessed if purified recombinant Mindin can function as a chemoattractant in vitro (Supplemental Figure 2, F and G). For this purpose, we sorted and cultured CD26^+^ and SCA1^+^ fibroblasts and seeded them in a Transwell chamber. Upon addition of Mindin to the lower chamber of the Transwell chamber, SCA1^+^ cells, but not the CD26^+^ cells, migrated in this assay (Figure 2C), indicating that the chemotactic response to Mindin is unique to the SCA1^+^ fibroblasts.

To gain insights into the molecular mechanisms underlying the promigratory effect of Mindin on fibroblasts, we performed gene set enrichment analysis (GSEA) on differentially upregulated genes (1,715 upregulated genes with *q* < 0.05; FC >1.5-fold; Supplemental Table 2) from Mindin-treated fibroblast RNA-Seq data (15). The analysis revealed significant enrichment in biological processes (Supplemental Table 3) associated with cell migration (41 genes) and positive regulation of cell migration (45 genes) (Supplemental Figure 2H). We created a sublist of 81 upregulated genes associated with these processes and reperformed GSEA. We found enrichment in processes associated with the cytoskeletal organization, cell adhesion, and ECM organization, along with the activation of integrin signaling and kinases involved in cell migration (Supplemental Figure 2I). Mindin is a known integrin ligand (31–33, 40–42), and we hypothesized that Mindin might activate the src family of kinases (SFK), given that the integrin-SFK axis has previously been reported to mediate fibroblast migration (43–45). To test whether Mindin exposure activates SFK in dermal fibroblasts, we probed for phospho-SRC levels via Western blot. We observed that Mindin was able to activate src family kinases within 15 minutes of treatment (Figure 2D and Supplemental Figure 2J). To assess whether the activation of src family kinases by Mindin is necessary for the migration of SCA1^+^ fibroblasts, we used 2 different SFK inhibitors — pp2 (pan SFK inhibitor) (46, 47) and KbSrc4 (a preferential inhibitor of c-SRC) (48). While the addition of pp2 to the Transwell chamber inhibited the migration of SCA1^+^ cells, this was not the case with KbSrc4 (Figure 2E). Given that KbSrc4 is more potent in inhibiting c-SRC over other SFK members such as FYN and YES (48), this suggested a differential role of SFK members in mediating the effect of Mindin. Thus, to delineate this further, we generated shRNA-based knockdown of *Src*, *Fyn*, and *Yes* kinases, which showed a 60%–80% reduction in RNA expression of *Src*, *Fyn*, and *Yes*, respectively (Supplemental Figure 2K). In a Transwell migration assay, SCA1^+^ cells transduced with nontargeting shRNA, Src shRNA, and Yes shRNA migrated in response to Mindin, but cells transduced with Fyn shRNA did not (Figure 2F). This indicated a nonredundant essential role of *Fyn* in the migration of SCA1^+^ fibroblasts downstream of Mindin.

We then investigated whether this phenomenon is characteristic of a physiological process such as wound healing. It has been previously shown that lower dermal fibroblasts (expressing SCA1) migrate into the wound bed at early stages followed by upper dermal fibroblasts in later stages (9). Given the evidence for the role of Mindin in fibroblast migration, we investigated whether Mindin is also important for the localization/recruitment of SCA1^+^ fibroblasts in wound healing. We wounded WT mice and quantified the expression of Mindin RNA in the wounded skin from day 1 to day 10 after wounding. We observed that Mindin expression starts increasing from day 3 after wounding, peaks at day 7, and decreased thereafter (Supplemental Figure 2L). We, therefore, stained the wounded skin for SCA1^+^ cells on day 7 and day 9 after wounding. Immunofluorescence staining on day 7 and day 9 after wounding skin revealed decreased numbers of SCA1^+^ cells localized in the wound bed in *Min*-KO mice compared with WT mice (Figure 2, G and H) consistent with a defect in SCA1^+^ fibroblast migration in the absence of Mindin. In line with the importance of lower dermal fibroblasts in the wound healing response (9)**,** the failure to recruit SCA1+ cells into the wound bed corresponded to a delay in wound closure during the postwounding day-7 to day-9 time frame (Figure 2I).

### Mindin induces an inflammatory phenotype in SCA1^+^ fibroblasts.

**Figure 3:**
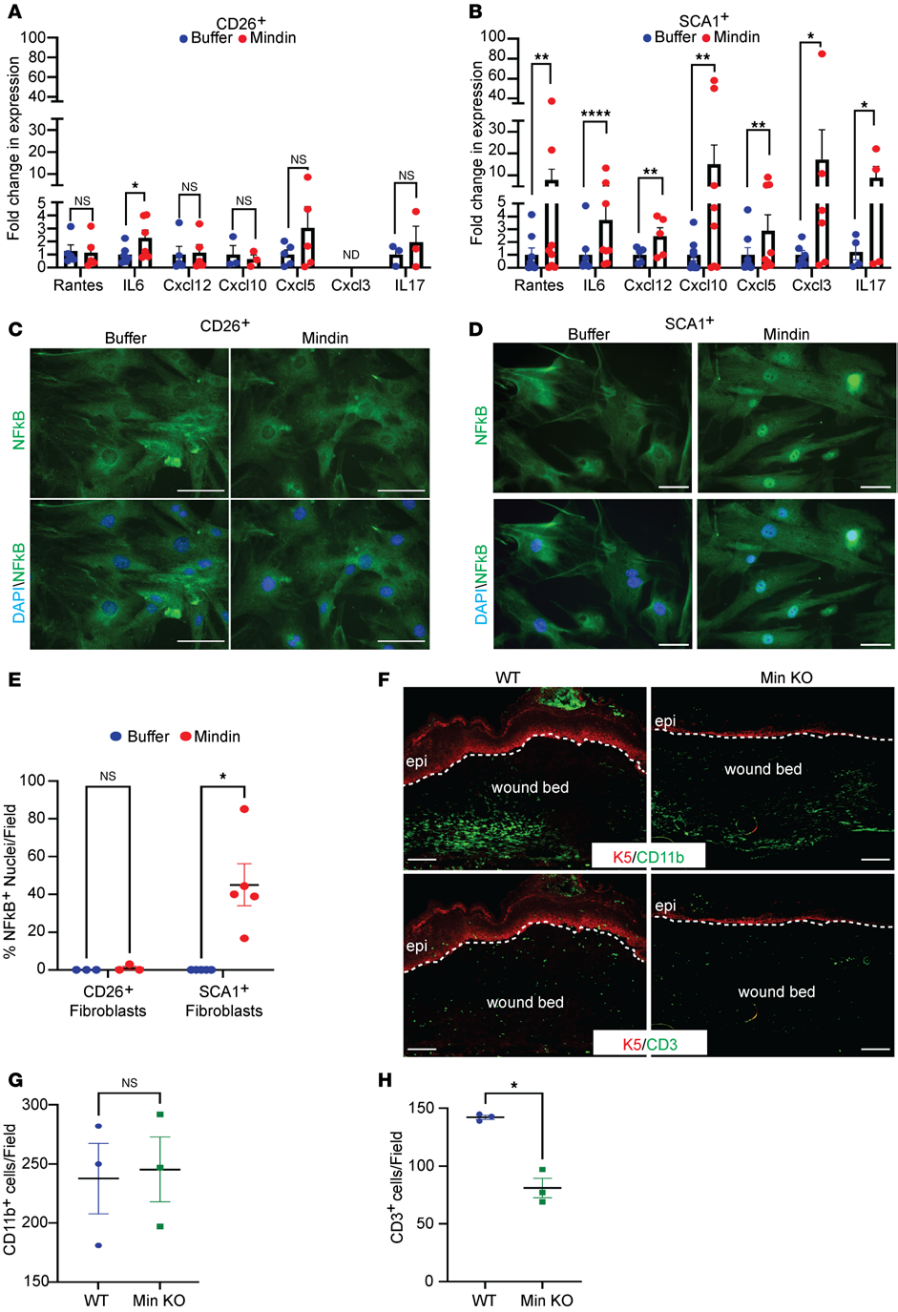
Mindin stimulates inflammatory cytokine production in SCA1^+^ fibroblasts. qPCR for expression of inflammatory cytokines from (**A**) CD26^+^ fibroblasts or (**B**) SCA1^+^ fibroblasts treated with either buffer or Mindin (*n* ≥ 4). Staining for NF-κB (green) and DAPI (blue) in (**C**) CD26^+^ fibroblasts or (**D**) SCA1^+^ fibroblasts treated for 1 hour with either buffer or Mindin (scale bar: 50 μm) and (**E**) the percentage of cells with NF-κB^+^ nuclei per field in CD26^+^ (*n* = 3) or SCA1^+^ (*n* = 5) fibroblast treated with either buffer or Mindin. (**F**) IF staining for K5 (red) and CD11b (top; green; macrophages) and CD3 (bottom; green; T cells) in WT and *Min*-KO skin sections after wounded day 7 (scale bar: 50 μm) and quantification of (**G**) CD11b^+^ and (**H**) CD3^+^ cells found in the wound bed (*n* = 3 mice). Data represent the mean ± SEM. *P* values were calculated by ratio paired *t* test (**A** and **B**) and Welch’s *t* test (**E**, **G**, and **H**). *P < 0.05, **P < 0.01, ****P < 0.0001; NS, *P* > 0.05. nd, not detected.

We have previously reported that there is robust inflammation in the *Snail*-Tg skin (36, 49), which is substantially decreased in the absence of Mindin (15). In addition, we now observe that purified Mindin is sufficient to induce inflammatory cytokine expression in fibroblasts in vitro (Supplemental Tables 2 and 3). These data are further supported by increased production of IL-1b and IL-6 cytokines upon treatment of fibroblasts with Mindin, as observed via ELISA (Supplemental Figure 3A). We then analyzed which fibroblast subpopulation responded to Mindin by launching an inflammatory response. Thus, we treated the sorted population with purified recombinant Mindin in vitro and quantified the expression of various cytokines, which were observed to be differentially upregulated in the RNA-Seq analysis of Mindin-treated fibroblasts. In CD26^+^ fibroblasts, Mindin did not cause significant upregulation of most inflammatory cytokines, but there was a small but significant increase in IL-6 expression (Figure 3A). On the other hand, Mindin-treated SCA1^+^ cells exhibited a robust upregulation of all the cytokines we analyzed (Figure 3B). Furthermore, CXCL-3, which was undetected in CD26^+^ fibroblasts, was expressed in SCA1^+^ fibroblasts and showed a robust increase in response to Mindin. These data indicate that Mindin is sufficient to strongly induce the expression of proinflammatory cytokines preferentially in SCA1^+^ fibroblasts. To validate if SCA1^+^ fibroblasts were indeed the dominant producers of inflammatory cytokines, we removed other CD26- and SCA1-expressing cells in the skin, such as hematopoietic cells (CD45) and endothelial cells (CD31). All cytokines tested except IL-6 were significantly increased in SCA1^+^ fibroblasts isolated from *Snail*-Tg skin (Supplemental Figure 3B). *Cxcl10* increased in both CD26^+^ and SCA1^+^ subpopulations; however, SCA1^+^ fibroblasts from *Snail*-Tg skin expressed higher levels of *Cxcl10* compared with CD26^+^ fibroblasts (Supplemental Figure 3B).

We then focused on the mechanism by which extracellular Mindin can stimulate cytokine gene expression. Mindin has been previously shown to activate the NF-κB pathway in renal cells (HK-2 cells) (26), and NF-κB is known to be important for inflammatory cytokine expression in multiple cell types, including fibroblasts (50–56). In line with this, we found that the NF-κB pathway was one of the KEGG pathways enriched in the GSEA in Mindin-treated fibroblasts (Supplemental Figure 3C and Supplemental Table 4). Moreover, the majority of SCA1^+^ cells demonstrated a nuclear translocation of NF-κB upon 1 hour of Mindin treatment (Figure 3, C–E, and Supplemental Figure 3, D and E).

Given our observation that there is a deficiency of SCA1^+^ cells in the wound bed on day 7 after wounding, we predicted that *Min*-KO wounds would also have a deficiency in recruiting immune cells into the wound bed. Consistent with this prediction, staining of day-7 wound sections revealed a reduced number of T cells in the wound beds of Mindin-null animals, though macrophages (detected by either CD11b or F4/80) were largely unaffected (Figure 3, F–H, and Supplemental Figure 3, F and G).

### Mindin induces collagen contraction in CD26^+^ fibroblasts in a c-Src–dependent manner.

**Figure 4:**
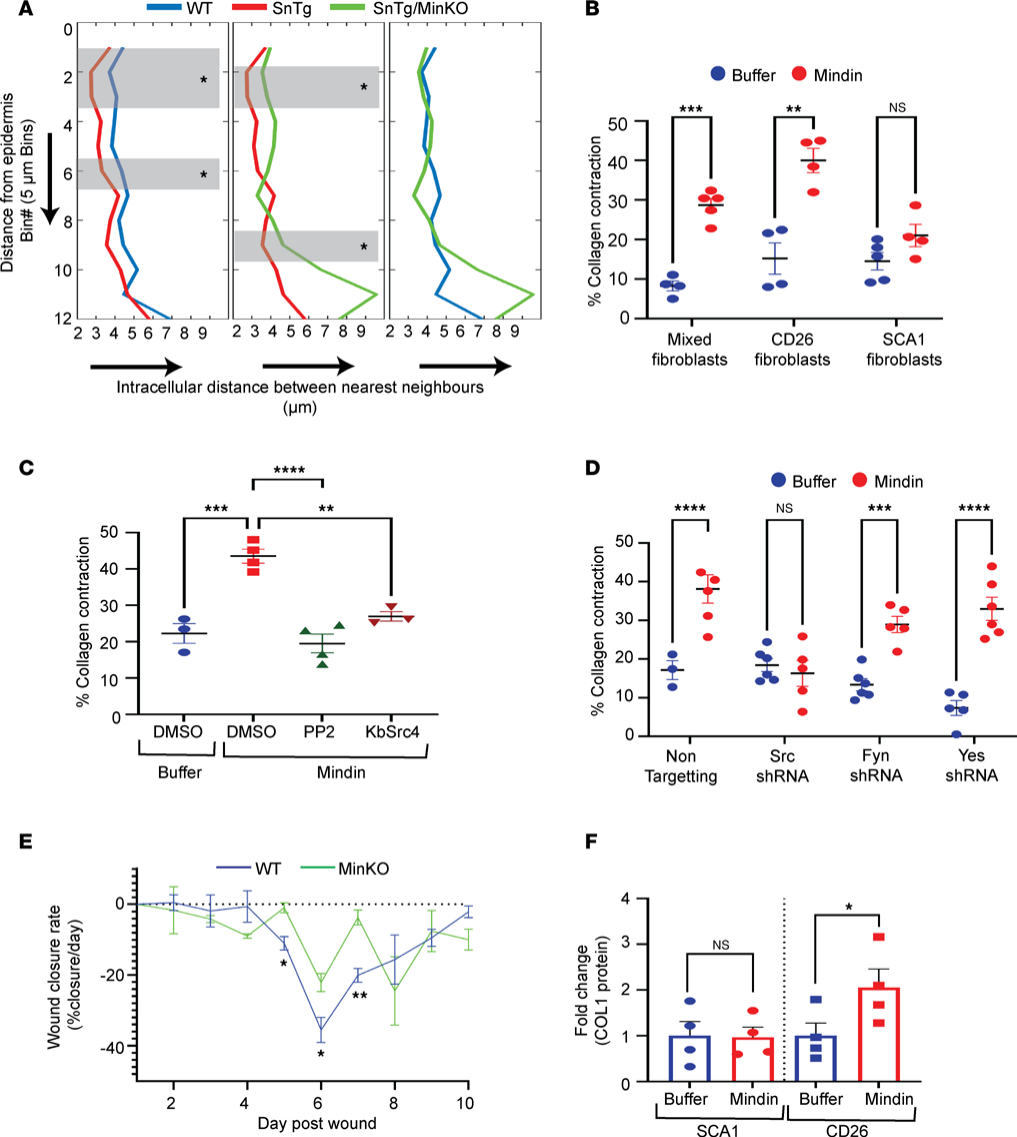
Mindin induces fibroblast contraction and collagen production in CD26^+^ fibroblasts. (**A**) Measurement of intracellular distance between 2 nearest CD26^+^ nuclei (*x* axis) as a function of distance below the epidermis (*y* axis, bin number below the epidermis; bin size = 5 μm) in WT, *SnTg*, and *SnTg/Min*-KO skin (*n* = 3). (The number of CD26^+^ cells counted >80 in each section. The region shaded in gray marks the bins where *P* < 0.05, calculated using Welch’s *t* test.) (**B**) Collagen contraction assay, showing percentage of contraction of collagen gels seeded with mixed, CD26^+^, or SCA1^+^ fibroblasts and treated with either buffer control or Mindin (*n* ≥ 4). (**C**) Effect of SFK inhibition on Mindin-induced collagen contraction. CD26^+^ fibroblasts were treated with either buffer control or Mindin along with DMSO, PP2, or KbSrc4 (*n* ≥ 3). (**D**) Effect of nontargeting (NT), *Src*, *Fyn*, or *Yes* shRNA on collagen contraction with CD26^+^ fibroblasts treated with either buffer control or Mindin (*n* ≥ 3). (**E**) Measurement of the rate of closure (slope) in WT and *Min*-KO mice. The slope was calculated as the percentage of closure of a given day – the percentage of closure on the previous day (*n* = 3 mice, 2 wounds per mouse). (**F**) Quantification of COL1 in buffer control or Mindin-treated CD26^+^ and SCA1^+^ fibroblasts, normalized to Lamin B1 (LAM) (*n* = 4). Data represent the mean ± SEM. *P* values were calculated by Welch’s *t* test (**B** and **E**), ratio-paired *t* test (**F**), 1-way ANOVA followed by Tukey’s post hoc analysis (**C**), and 2-way ANOVA followed by post hoc Šídák’s multiple comparisons test (**D**). *P < 0.05, **P < 0.01, ***P < 0.001, ****P < 0.0001; NS, *P* > 0.05.

Though there was not an obvious perturbation in the localization of CD26^+^ fibroblasts, a qualitative change in both a denser packing and a more uniform orientation of papillary fibroblasts was observed in the *Snail*-Tg skin, which was lost in the absence of Mindin (Supplemental Figure 4A). To test whether there is denser packing of the papillary fibroblasts, the distance between 2 neighboring CD26^+^ cells was measured and plotted as a function of the distance from the epidermis (Figure 4A). In agreement with the qualitative observation, the quantification revealed a significant reduction in the intracellular distance between neighboring CD26^+^ cells in *Snail*-Tg mice closer to the epidermis. Nevertheless, in *Snail*-Tg*/Min*-KO mice, the dense packaging of CD26^+^ cells was attenuated. Many factors can influence the density of cells in the dermis, one of which is the increased number of CD26^+^ cells in the *Snail*-Tg background. However, there was no difference in the proportion of papillary fibroblasts in the WT versus *Snail*-Tg skin (Supplemental Figure 1, B–E). Another possibility is that myofibroblast contraction may result in the compaction of the matrix resulting in an increased localized density of cells. Furthermore, fibroblasts in contracting gels become parallelly aligned and are closely packed together (57). Therefore, we hypothesized that CD26^+^ fibroblasts might differentiate to contractile myofibroblasts in the *Snail*-Tg skin in a Mindin-dependent fashion. To test this hypothesis, heterogeneous fibroblasts and FACS-sorted SCA1^+^ and CD26^+^ fibroblasts were embedded in collagen gels and treated with Mindin. 72 hours after treatment, we measured the area of the treated gels and found that Mindin can promote collagen contraction in gels seeded with either mixed fibroblasts or CD26^+^ fibroblasts (Figure 4B and Supplemental Figure 4B). However, this was not the case for SCA1^+^ fibroblasts.

We then investigated the mechanism by which Mindin can induce contraction in CD26^+^ fibroblasts. The SFK has been shown to be involved in the regulation of myofibroblast differentiation and contraction downstream of TGFb1 (58). The integrin-src signaling axis has been implicated in the regulation of RhoA-ROCK activation, which can affect stress fiber formation and contractile activity via myosin light chain phosphorylation (59). Given our observation that Fyn kinase drives the migration of SCA1^+^ cells in response to Mindin, we investigated whether SFKs are also necessary for Mindin-induced contraction of CD26^+^ cells. Both the pan-SFK inhibitor PP2 as well as the c-SRC–specific inhibitor KbSrc4 blocked Mindin-mediated contraction (Figure 4C and Supplemental Figure 4C). To further access which member of the SFK was required for the contraction of CD26^+^ cells, we knocked down *Src*, *Fyn*, and *Yes* kinases by transduction of specific shRNAs. The transduced cells were embedded in collagen gels and treated with Mindin. While CD26^+^ fibroblasts transduced with nontargeting, Fyn, or Yes shRNA contracted the collagen gels in response to Mindin, reduction of c-SRC inhibited the Mindin-mediated contraction (Figure 4D and Supplemental Figure 4D). This indicated that specific src family kinase members are differentially utilized for different aspects of fibroblast activation.

One of the physiological roles of fibroblast contraction is its contribution to the contraction of the wound bed, which aids in wound closure. Since we observed a delay in wound healing in Mindin-null animals (Figure 2I), we calculated the rate of wound closure (as measured by the percentage of closure/day) in WT and *Min*-KO wounds as a proxy for in vivo tissue contraction. Our results show that contraction in WT mice starts on day 5 and continues until day 7 after wounding. Interestingly, the magnitude of the contraction is lower in the *Min*-KO animals (Figure 4E). However, unlike that for SCA1^+^ cells, we did not observe any deficiency in the recruitment of CD26^+^ fibroblasts in the wound bed of *Min*-KO animals (Supplemental Figure 4, E and F). This indicates that, while Mindin is not required for the migration and recruitment of CD26^+^ cells, it plays a role in the contraction of the wounds.

Apart from contraction, another consequence of fibroblast activation is the increase in collagen production. Thus, to test if Mindin can affect collagen production, we treated the sorted fibroblasts with Mindin and measured the levels of collagen (COL1A1 and COL1A2) via Western blot (Figure 4F and Supplemental Figure 4G). Only CD26^+^ cells exhibited an increase in the amount of COL1 levels upon treatment with Mindin. Though COL1 is the most abundant collagen and contributor to tissue fibrosis, there are other collagens that play important roles in this pathology such as COL3, COL4, COL5, and COL7 (60, 61). The type of collagen upregulated may differ by the ligand and fibroblast subtype. Therefore, to test if Mindin differentially regulates the expression of collagen subtypes in CD26^+^ and SCA1^+^, we measured the mRNA expression level of these collagens via qPCR. *Col1a2*, *Col3a1*, and *Col5a* were significantly upregulated in Mindin-treated CD26^+^ cells (Supplemental Figure 4H). Interestingly, *Col3a1* was also significantly increased in Mindin-treated SCA1^+^ cells (Supplemental Figure 4H). These results reveal how different fibroblast subpopulations contribute in a complementary fashion to the overall increase in the bulk amount of collagen proteins in the fibrotic tissue.

### Mindin promotes a CAF-associated self-renewal promoting capability in CD26^+^ fibroblasts.

The mesenchymal compartment of fibrotic tissues shares remarkable similarities with the stroma surrounding solid tumors (19, 20). We thus hypothesized that Mindin might play a role in the generation of CAFs, which are crucial in tumorigenesis (20, 22). Supporting the potential role of Mindin in other fibrotic scenarios, such as solid tumors, there are reports of Mindin being upregulated in many cancers, where it is proposed as a potential diagnostic and prognostic biomarker (31–35). Furthermore, GSEA using Mindin-upregulated genes in fibroblasts revealed a significant enrichment of disease terms in the DisGeNET database for inflammatory and fibrotic diseases as well as cancers (Supplemental Figure 5, A and B).

**Figure 5:**
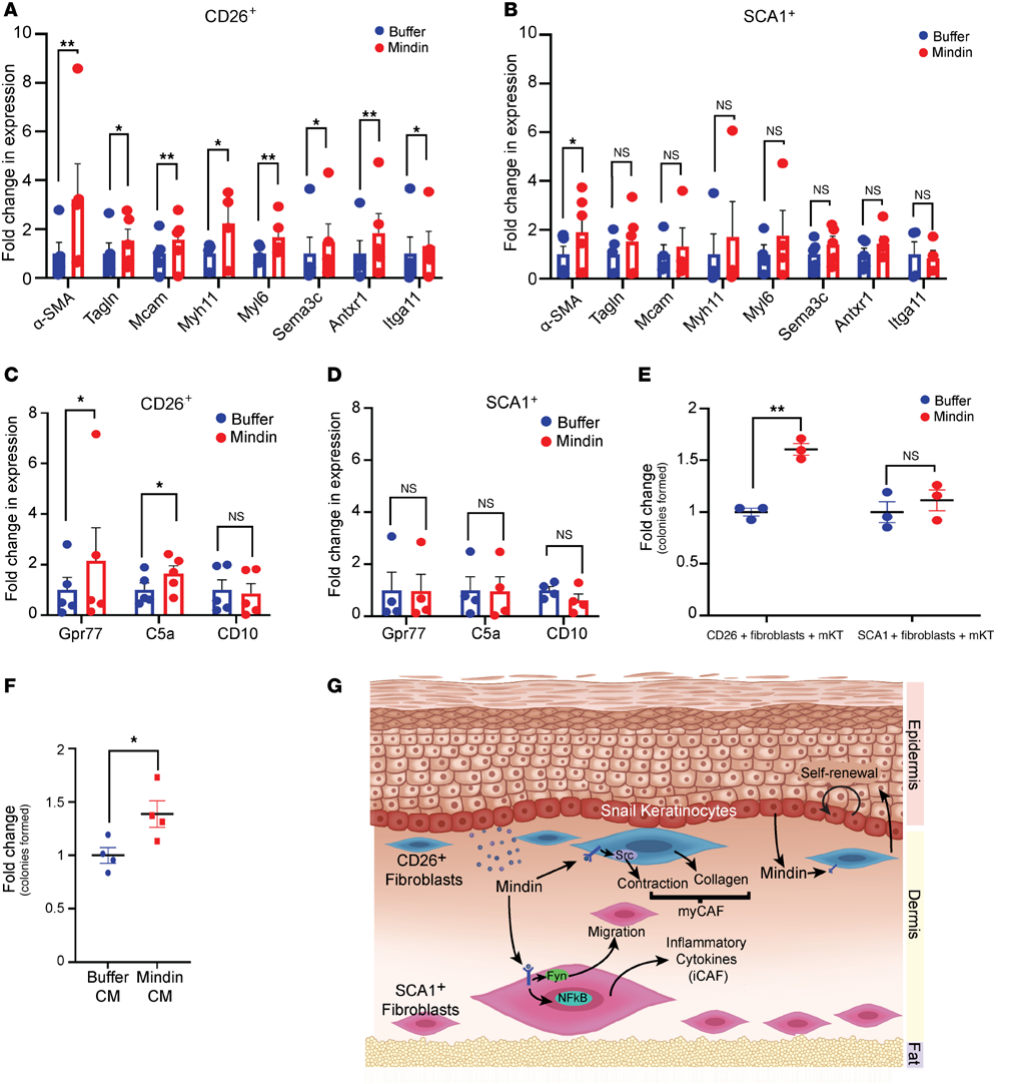
Mindin promotes CD26^+^ fibroblasts to adopt a CAF phenotype. qPCR for expression of signature genes of myCAFs in (**A**) CD26^+^ fibroblasts or (**B**) SCA1^+^ fibroblasts treated with either buffer or Mindin (*n* ≥ 4). Expression of genes that are associated with stem cell renewing CAFs in (**C**) CD26^+^ (*n* = 6) and (**D**) SCA1^+^ (*n* = 4) fibroblasts treated with either buffer or Mindin measured by qPCR. (**E**) Colony formation assay of primary mouse keratinocytes (mKT) cocultured with CD26^+^ or SCA1^+^ fibroblasts pretreated with either buffer of Mindin for 24 hours (*n* = 3). (**F**) Colony formation assay of primary mouse keratinocytes cultured with conditioned media (CM) collected from CD26^+^ fibroblasts treated with either buffer or Mindin (*n* = 4). Data represent the mean ± SEM. *P* values were calculated by ratio paired *t* test (**A**–**D**) and Welch’s *t* test (**E** and **F**). *P < 0.05, **P < 0.01; NS, *P* > 0.05. (**G**) Model of differential effects of Mindin on distinct subpopulations of dermal fibroblasts.

As shown in Figure 3, A and B, Mindin differentially primes SCA1^+^ fibroblasts to adopt an inflammatory phenotype, consistent with iCAFs, which have also been shown to express *IL6*, *CCL5*, *CXCL12*, *CXCL10*, and *CXCL3* (22, 62, 63). Similarly, treatment with Mindin induced CD26^+^ fibroblasts to become more contractile and secrete elevated levels of collagen (Figure 4, B and F), which is characteristic of a myCAF phenotype (22). To test whether this is supported by molecular markers, we analyzed the expression levels of signature genes of myCAFs (*aSma*, *Tagln*, *Mcam*, *Myh11*, *Myl6*, *Antrx1*, *Sema3c*, *Itga11*) (62, 63) in Mindin-treated CD26^+^ and SCA1^+^ fibroblasts. While α-SMA expression was increased in both subpopulations treated with Mindin, other reported myCAF markers were significantly elevated only in Mindin-treated CD26^+^ cells (Figure 5, A and B). Furthermore, it has been shown that CD10, C5a, and GPR77 are signatures of a subset of CAFs in patients with breast and lung cancer (64). Interestingly, only CD26^+^ fibroblasts increased expression levels of *Gpr77* and C5a upon Mindin treatment, though there was no effect on CD10 expression (Figure 5, C and D). C5a binds to its receptor GPR77, which activates NF-κB in a positive feedback loop to further increase the expression of *GPR77* (64). In line with this, we found that prolonged treatment of CD26^+^ fibroblasts with Mindin can activate NF-κB signaling in these cells (Supplemental Figure 5, C and D).

An important function of myCAFs is the maintenance of cancer stem cells and chemoresistance, which is associated with poor prognosis (64). We tested whether Mindin-treated fibroblasts are functionally equivalent to myCAFs and, in particular, capable of promoting the self-renewal of epithelial progenitor/stem cells. For this purpose, we cocultured primary mouse epidermal keratinocytes (mKTs) with CD26^+^ or SCA1^+^ fibroblasts pretreated with either buffer or Mindin and measured self-renewal using a colony formation assay. Only Mindin-treated CD26^+^ fibroblasts were able to increase colony formation of mKTs (Figure 5E). Furthermore, culturing mKTs in conditioned media collected from Mindin-treated CD26^+^ fibroblasts was sufficient to increase colony formation (Figure 5F), indicating a role of molecular crosstalk between CD26^+^ dermal fibroblasts and keratinocytes via soluble factors.

## Discussion

Our data reveal Mindin as a modulator of heterogeneous dermal fibroblasts that drive cutaneous fibrogenesis (Figure 5G). We have shown that Mindin, secreted by *Snail*-Tg keratinocytes (18), elicits functional responses in resident subpopulations of fibroblasts in the skin. Mindin mediated the migration of SCA1^+^ fibroblasts via Fyn kinase and increased inflammatory cytokine production in these cells. Conversely, Mindin induced a more contractile phenotype in CD26^+^ fibroblasts and increased collagen I production. However, Mindin utilized c-Src to mediate this effect in papillary fibroblasts. Interestingly, the effect of Mindin on SCA1^+^ and CD26^+^ fibroblasts endowed them with features of iCAFs and myCAFs, respectively. Additionally, Mindin-treated CD26^+^ fibroblasts could promote self-renewal of epithelial cells, akin to the role of myCAFs in maintaining cancer stem cells. These data are consistent with the notion that CAFs can be derived from resident fibroblasts within the tissue (65, 66). Furthermore, we have previously analyzed published datasets of various fibrotic models and observed that Mindin was also overexpressed in bleomycin-induced fibrosis in skin and lungs, a unilateral ureteral obstruction kidney fibrosis model, and a high-fat diet–induced nonalcoholic steatohepatitis (NASH) model (15). This suggests that the role of Mindin may not be limited to *Snail*-Tg mice but could extend to other fibrotic mouse models and tissues. Fibroblast heterogeneity has also been reported in the human skin. Single-cell RNA-Seq has revealed that human dermal fibroblasts can be categorized into 4 subgroups — namely, mesenchymal, inflammatory, secretory papillary, and secretory reticular subgroups (67). In fibrotic conditions, such as keloids and SSc, specific markers for each subpopulation are differentially regulated (68). Notably, we found a significant overlap with the markers of these subpopulations in Mindin-treated human dermal fibroblasts (Supplemental Figure 6A). We found that the spatial organization of SCA1^+^ cells is perturbed in the *Snail*-Tg background and have attributed Mindin-mediated migration as an underlying cause. Besides cellular migration, another possible explanation is the transdifferentiation of CD26^+^ cells into SCA1^+^ fibroblasts. One prediction of transdifferentiation from CD26^+^ to SCA1^+^ fibroblasts is the presence of a transitional cell state, where cells would express both markers simultaneously, making them double positive. However, both WT and *Snail*-Tg skin contained only a small percentage of fibroblasts that were double positive for CD26 and SCA1 (Supplemental Figure 1, C and F), which cannot explain the increased number of SCA1^+^ cells at the dermal-epidermal junction in the transgenic animal. Additionally, treatment with Mindin did not induce expression of SCA1 in CD26^+^ fibroblasts or vice versa (Supplemental Figure 6B). It should be noted that SCA1 is also expressed on endothelial cells (marked by CD31) and some hematopoietic cells (marked by CD45). However, CD45^–^CD31^–^SCA1^+^ cells were more predominant in the total cell population and showed an increase in *Snail*-Tg skin, while CD45^+^CD31^+^SCA1^+^ did not significantly change in the *Snail*-Tg skin (Supplemental Table 5). This makes the hematopoietic compartment or endothelial cells an unlikely source for the increased proportion of SCA1^+^ cells. SCA1^+^ fibroblasts have been reported to express preadipocyte markers and contribute to the maintenance of adipose tissue homeostasis (8–10). A functional consequence of relocating the SCA1^+^ fibroblasts from the lower to the upper dermis is the deprivation of a potential source of adipose tissue that lies within the lower dermis. Consistent with this, in the adult *Snail*-Tg skin, there is a near-total absence of dermal white adipose tissue that is replaced by collagen (15).

One notable observation was the increase in the number of SCA1^+^ fibroblasts in the *Snail*-Tg skin (Supplemental Figure 1, A and D) without an increase in proliferation (Supplemental Figure 6C). This increase may be possible through the inhibition of SCA1^+^ fibroblasts differentiating into other lineages. As noted earlier, under homeostatic conditions, SCA1^+^ fibroblasts differentiate to maintain adipose tissue homeostasis (9). However, β-catenin stabilization in SCA1^+^ fibroblasts has been shown to inhibit their differentiation into adipocytes, thereby promoting fibrosis (8). Our results indicate that Mindin treatment activates β-catenin signaling in SCA1^+^ fibroblasts in vitro, as measured by an increase in the β-catenin target gene *Axin2* (Supplemental Figure 6D). This suggests that the increase in SCA1^+^ cells in the *Snail*-Tg skin is at least partly due to an accumulation of cells that do not otherwise differentiate into adipocytes. However, inhibition of β-catenin using the iCRT5 inhibitor (69) did not inhibit Mindin-mediated migration (Supplemental Figure 6E). Nevertheless, it cannot be ruled out that other fibroblast subpopulations, such as Dlk^+^SCA1^–^ reticular fibroblasts, which have been shown to contribute to all compartments of the dermis (9), do not contribute to the SCA1^+^ population in *Snail*-Tg mice.

It has been shown that lower dermal fibroblasts are the major source of fibrous collagen during homeostasis and physiological repair (37, 38). Nevertheless, we observed that it was the papillary fibroblasts that increased the levels of Col1 in response to Mindin. This suggests that in fibrotic scenarios CD26^+^ cells can be induced to provide a substantial amount of ECM proteins. This is consistent with the report by Rinkevich et al. (11) that En1^+^-derived CD26^+^ fibroblasts constitute the major population with fibrogenic potential and a CD26 inhibitor inhibits scarring.

In addition to stimulating a local contracture, the ordered packaging of CD26^+^ papillary fibroblasts is also consistent with the alignment of myCAFs observed immediately adjacent to carcinomas (70). This packaging can arise from the contractile forces exerted on the matrix by myCAFs and the remodeling of the local ECM (70). Along with showing features consistent with myCAFs, the papillary fibroblasts are also located in a histological position consistent with the origins of myCAFs.

Altogether, these findings provide what we believe to be new insights into the heterogeneous regulation of fibrosis-associated and CAFs. Many interesting questions remain to be resolved. For instance, the differential response of fibroblast subpopulations corresponding to different members of SFKs may hint at the differential expression of cognate Mindin receptors in these cells. These differences may be at the level of surface expression of distinct integrin pairs (which have been shown to act as receptors for Mindin on macrophages, T cells, and colorectal cancer) (40, 41, 71) and/or different cytoplasmic machinery downstream of the receptors. The differences may also arise owing to distinct levels and/or localization of SFK members or their adaptor molecules in different fibroblasts. Our transcriptomic data (Supplemental Table 2) did reveal differential expression of Fyn upon Mindin treatment. These possibilities should thus be explored in future studies. Furthermore, there are additional fibroblast subpopulations that may contribute to fibrotic tissue and were not addressed in this study. These include Dlk^–^SCA1^–^ reticular fibroblasts, pericytes, fibrocytes, and mesenchymal stem cells. Moreover, further refinement of these SCA1^+^ and CD26^+^ subpopulations and their responses to different profibrotic stimuli may shed light on the pathophysiology of fibrosis and the tumor stroma. For example, myCAFs have been reported to be a double-edged sword, capable of both promoting (22) and restraining tumors (72). Thus, it would be interesting to determine if refinement of the CD26^+^ subpopulation can explain the 2 opposing phenotypes or if the same subpopulation can switch the phenotype in response to different microenvironmental compositions.

## Methods

### Sex as a biological variable.

Our study examined both male and female animals, and similar results were observed for both sexes.

### Animal studies.

C57BL/6 (WT) mice were obtained from The Jackson Laboratory, *Min*-KO mice were obtained from You-Wen He (Duke University, Durham, North Carolina, USA), and the *Snail*-Tg mouse was engineered as described earlier (14). The K14-*Snail*-Tg/*Min*-KO mouse was developed by breeding *K14-Snail*-Tg and *Min*-KO mice.

### Cell culture.

Primary newborn dermal fibroblasts (mixed fibroblasts) isolation was performed as described earlier (36) from C57BL/6 P2-P3 pups. Human primary dermal fibroblasts were procured from ScienCell Research Laboratories (catalog 2320). All fibroblasts were cultured in DMEM high-glucose media with 10% FBS. All experiments were performed on fibroblasts in between passages 2 and 5. Primary mKTs were harvested from the P2/P3 epidermis and cultured in low-calcium E-media to maintain an undifferentiated proliferating state as previously described (73, 74). Further details on the sorting and culture of CD26 and SCA1 fibroblasts are provided in Supplemental Methods.

### Mindin purification and treatment.

Histidine-tagged Mindin was purified from conditioned media collected from CHO-Mindin cells (which were a gift from You-Wen He, Duke University) using Ni-NTA beads (Thermo Fisher Scientific) according to the manufacturer’s protocol. Buffers with varying strengths of imidazole were made in 10 mM Tris and 300 mM NaCl (pH = 8) for washing and elution. The purified Mindin (10 mL) was dialyzed for 3 rounds in 1 L dialysis buffer (10 mM tris, 20 mM NaCl, pH = 8), concentrated using a 10 kDa Centricon concentrator, and filtered with a 0.2 μm syringe filter. Silver staining and Western blot using Mindin antibody (Santa Cruz, SC49050, at dilution 1:100, and Jackson ImmunoResearch, 705-035-147, secondary, at dilution 1:200) were performed to assess the purity and confirm the purification of Mindin. The purified recombinant Mindin was used at a concentration of 80–200 ng/mL in all treatments. All treatments were done in serum-free conditions unless otherwise stated.

### Fluorescence-activated cell analysis.

The detailed flow cytometry method is described in Supplemental Methods.

### Lentiviral transductions.

All lentiviral production and transductions were done in a BSL-2 facility in accordance with inStem Institutional Biosafety Committee–approved protocols. The method for lentiviral particle generation and the shRNA-pZip-mEf1a plasmids for *c-Src*, *Fyn*, and *Yes* shRNA (procured from transomics) have been described earlier (21). The titer of the virus that yielded greater than 50% GFP^+^ cells was used to transduce sorted SCA1^+^ or CD26^+^ fibroblasts after the first passage, at 70%–80% confluency. 72 hours after infection, the virus-containing media was removed, and fresh media with 1 μg/mL puromycin was added to enrich transduced cells. Plates showing greater than 90% GFP^+^ cells were used for further experiments.

### Transwell migration assay.

The detailed methodology for Transwell migration assay is described in Supplemental Methods.

### Collagen contraction assay.

Collagen contraction assay was done as previously described (15, 75) with newborn dermal fibroblasts or SCA1^+^, CD26^+^, or shRNA-transduced fibroblasts. The detailed protocol is described in Supplemental Methods.

### Western blot.

Cells were serum-starved overnight before treatment and were treated with either buffer or Mindin in a serum-free medium for 15 minutes. Cells were lysed in RIPA buffer and were loaded after the addition of Laemmli buffer. The membranes were probed for phosphorylated SRC (CST, 2101) and then with total SRC (CST, 2123) after stripping. For assessment of collagen I, serum-starved cells were treated with either buffer or Mindin for 48 hours. Cells were lysed in RIPA, and the lysates were loaded in the gels along with Laemmli buffer. The membranes were probed with anticollagen antibodies (Abcam, ab21286) and Lamin B (Abcam, AB16048). The HRP-labeled secondary antibodies (Jackson ImmunoResearch, 305-035-003) were used at 1:3,000 dilution. Blots were developed on an ImageQuant LAS4000, and bands were quantified using Fiji software (ImageJ [NIH]).

### Cell localization analysis.

The detailed methodology for cell localization analysis is described in the Supplemental Methods.

### Nearest-neighbor analysis.

The detailed methodology for nearest-neighbor analysis is described in the Supplemental Methods.

### Colony formation.

The detailed methodology for colony formation is described in the Supplemental Methods.

### Wound healing.

Two excisional wounds (separated by 1–1.5 cm) were created on the middorsal skin of the anesthetized 3- to 4-month-old mice. Images were taken from day 1 to day 10 after wounding. Percentage wound closure was calculated as *C* = (1 – *W_n_*/*W*_1_) × 100, where *C* is the percentage of wound closure, *W_n_* is the wound area on day *n*, and *W*_1_ is the wound area on day 1. The slope or rate of wound closure was determined as percentage of closure/day, *R* = *C_n_*–*C*_n–1_, where *C_n_* is the percentage of closure on a given day *n* and *C*_n–1_ is the percentage of closure on the previous day. Mice were sacrificed, and wounds were harvested on day 0 (unwounded) and days 3, 5, 7, 9, and 10. (wound closed). Tissue was stored in RNA later (Sigma) (for gene expression analysis) or embedded in OCT (Leica Biosystems) for immunofluorescence.

### NF-κB nuclear localization.

Cells were seeded in a 96-well dish (10,000 cells/well) and were serum-starved overnight 24 hours later, followed by treatment with either buffer or Mindin. After treatment cells were fixed with 4% PFA, permeabilized, and stained with NF-κB (Santa Cruz, SC372) antibody at 1:200 dilution. A secondary antibody (Jackson ImmunoResearch, 711-545-152) was used at 1:200. A DAPI stain was used to mark nuclei. Images were captured with an Olympus IX73 microscope.

### Immunofluorescence of skin sections.

Skin tissues were fixed and sectioned as previously reported (15) and probed with the following antibodies diluted 1:200: K5 (as previously described by CJ’s lab in Rana et al., 2023, ref. 15; Badarinath et al., 2022, ref. 18; Pincha et al., 2018, ref. 49; Nakasaki et al., 2015, ref. 36); SCA1 (R&D Systems, AF1226); CD26 (R&D Systems, AF954); CD11b (Abcam, ab8878); CD3 (ebiosciences, 14-0032-85), and F4/80 (ebiosciences, 14-4801-81). α-SMA (Abcam ab5694) was used at 1:50 dilution. Secondary antibodies (Jackson ImmunoResearch, 711-545-152, 711-575-152, 712-545-150, 712-575-150, 703-545-155, and 705-545-147, and Invitrogen, A11055, A11057, and A21208) were used at a 1:200 dilution. DAPI stain was used to mark nuclei. Images were captured with an Olympus IX73 microscope.

### GSEA.

A gene list was created using upregulated genes with adjusted *P* values (*q*) of less than 0.05 and fold changes of more than 1.5 from the RNA-Seq dataset of Mindin-treated human dermal fibroblasts described earlier (15). The Database for Annotation, Visualisation and Integrated Discovery (DAVID; https://davidbioinformatics.nih.gov/home.jsp) was used for GSEA (76–78). GOTERM_BP_Direct (79, 80) was used to visualize enriched biological processes, KEGG (https://www.genome.jp/kegg/) pathways (81) were used for identifying associated signaling pathways, and the DisGeNET database (https://disgenet.com/) was used for identifying the enrichment in associated diseases (82). Marker genes for different fibroblasts in keloids were extracted from Deng et al., 2021 (68). Gene overlap analysis was performed and Venn diagrams were created using Venny 2.0 (83).

### Gene expression.

Total RNA was extracted from cells treated with either buffer or Mindin for 16 hours using TRIzol Reagent (TaKaRa, Thermo Fisher Scientific), and cDNA was synthesized using Superscript III (Thermo Fisher Scientific). The quantitative PCR (qPCR) was performed using Power SYBR Mix (Life Technologies, Thermo Fisher Scientific) in a Bio-Rad CFX384 machine. *Gapdh* or Actin (*Actb*) expression was used as a reference for normalization. The primer sequences used are listed in Supplemental Table 6.

### ELISA.

The ELISA for IL-6 (Sigma, RAB0306) and IL-1β (R&D Systems, DLB50) was performed on dermal fibroblasts treated with Buffer, rMindin, conditioned media, or IL-1α (as positive control) according to the manufacturer’s protocols.

### Statistics.

Welch’s 2-tailed *t* test was used for the comparison of the 2 groups. Ratio-paired 2-tailed *t* test was used for comparison of fold changes in paired data. One-way ANOVA followed by Tukey’s post hoc analysis was used for comparing 3 or more groups. Two-way ANOVA followed by post hoc Šídák’s multiple comparisons test was used to compare 3 or more groups over multiple conditions. GraphPad Prism 6 was used for all statistical analyses. *P* values for overlap between gene lists were calculated using a hypergeometric test available at http://nemates.org/MA/progs/overlap_stats.html The data are represented as the mean ± SEM. *P* values of less than 0.05 were considered significant.

### Study approval.

Animal work conducted at the National Centre for Biological Sciences/inStem Animal Care and Resource Centre was approved by the inStem Institutional Animal Ethics Committee following the norms specified by the Committee for Control and Supervision of Experiments on Animals (government of India). All experimental work was approved by the inStem Institutional Biosafety Committee.

### Data availability.

RNA-Seq data for Mindin-treated fibroblasts used in this study are deposited in NCBI SRA database BioProject (accession ID PRJNA846577; https://www.ncbi.nlm.nih.gov/bioproject/PRJNA846577). Values for all data points in graphs are reported in the Supporting Data Values file.

## Supplementary Material

Supplemental data

Unedited blot and gel images

Supplemental tables 2-4

Supporting data values

## Version 1 12/31/2024

In-Press Preview

## Version 2 02/10/2025

Electronic publication
