# Supplementary material for: Mindin regulates fibroblast subpopulations through distinct Src family kinases during fibrogenesis
Source: JCI Insight. 2024 Dec 31;10(3):e173071. doi: 10.1172/jci.insight.173071 (PMC11948575; doi:10.1172/jci.insight.173071)
Supplement: Supplemental data [file jciinsight-10-173071-s144.pdf]

**Supplementary methods**

**Cell sorting**

For $CD26^+$ and $SCA1^+$ fibroblasts, the dermis was separated from the skin of P2 or P3 mice by incubating in 1 mg/ml dispase for 1 hour at 37 °C, followed by digestion in 2.5 mg/ml collagenase IV (Gibco) for 1 hour at 37 °C. Dermal cell suspensions from 4–5 P2/P3 pups were pooled and stained with SCA1-Alexa Fluor 700 (R&D FAB1226N; 1:30 µl) and CD26-PE (FAB9541P; 1:10 µl) for 30 minutes on ice. $CD26^+SCA1^-$ ($CD26^+$) and $SCA1^+CD26^-$ ($SCA1^+$) cells were sorted directly from this dermal suspension, without any prior culturing, using BD-FACSAria Fusion or ARIA III. Laser delay and area scaling were performed using BD Sphero beads, and drop delay was adjusted using ACD beads. An unstained sample was used to set the voltages. An SSC-A and FSC-A plot was used to gate the cell population, and an FSC-A and FSC-H plot was used to create a diagonal gate to select single cells. A Propidium iodide (PI; Sigma P4864) stained tube was used to identify the dead cell population, and the experiment proceeded only if the dead cell population was less than 15% of all events and less than 10% of gated cells. Single-stained CD26 and SCA1 samples were then recorded. A quadrant gate in the CD26-PE and SCA1-AF700 channels was used to perform compensation by ensuring that the X and Y median in the lower left quadrant of the unstained and single-stained tubes matched the X and Y median of the upper left and lower right quadrants in the single-stained tubes. The co-stained tube was then used to sort $CD26^+SCA1^-$ and $SCA1^+CD26^-$ cells. The sorted cells were pelleted at 100g and cultured in 10% FBS DMEM medium for two passages before use in experiments.

For RNA isolation from $SCA1^+$ and $CD26^+$ cells directly from the dermis of WT and *Snail transgenic* skin, the dermal cell suspension from the mice was stained with SCA1-AF700, CD26-AF405 (R&D FAB9541V), CD45-PE (MACS Miltenyi Biotec 130-102-781), and CD31-PE (MACS Miltenyi Biotec 130-102-971) antibodies. Sort settings were configured as described above. The co-stained cells were then used to sort $CD45^-CD31^-CD26^+SCA1^-$ and $CD45^-CD31^-SCA1^+CD26^-$ cells. Tubes with sorted cells were centrifuged at 100g, and the pellet was resuspended in Trizol for RNA isolation.

**Collagen Contraction assay:**

Rat tail collagen (MilliporeSigma; 08-115) was dissolved in 0.1% acetic acid to create a 3 mg/ml stock solution. A cell suspension of NBDF, $SCA1^+$, $CD26^+$, or shRNA-transduced fibroblasts was prepared in 0.5% serum media with 150,000 cells/ml. Cells and gels were mixed by combining collagen stock and cell suspension in a 1:2 ratio (final concentration: 1 mg/ml collagen, 100,000 cells/ml), along with the simultaneous addition of an appropriate amount of 1 M NaOH (predetermined via titration, approximately 8–10 µl/ml). Cell-free gels were prepared by mixing collagen stock and media without cells at a 1:2 ratio. The mixture was immediately added to either 24-well (500 µl/well), 48-well (250 µl/well), or 96-well (100 µl/well) plates and incubated at 37 °C for 1 hour to solidify. After solidification, serum-free media (in the same volume as the gel), along with either buffer or Mindin, was added to each well. For inhibitor assays, PP2 (10 µM) or KB SRC 4 (10 µM) was added along with buffer or Mindin. The gels were detached from the walls using a pipette tip and kept at 37 °C. After 72 hours, the experiment was terminated, gels were stained with crystal violet solution for contrast enhancement, and images were acquired for quantification. The percentage of contraction was calculated as $C=(1-A_t/A_{nc})*100$, where $A_t$ is the area of collagen gel containing cells and with treatments and $A_{nc}$ is the area of collagen gel with no cells added, as measured 72-hours post the detachment step.

**Flow cytometry:**

P9 pups were sacrificed, and all hair was removed using Veet hair removal cream. The dermal cell suspension was prepared by separating the dermis from the epidermis in 1 mg/ml Dispase overnight at 4 °C. The dermis was then digested in 2.5 mg/ml collagenase IV (Gibco) for 1 hour at 37 °C. The dermal cell suspension was filtered through a 40-micron strainer, centrifuged, and resuspended in FACS buffer (0.1% FBS in PBS). Cells were stained with LIVE/DEAD™ Fixable Lime (506) Viability dye (Thermo L34989), SCA1-AF700 (R&D FAB1226N; 1:30 µl), and CD26-PE (R&D FAB9541P; 1:10 µl) or their respective isotype controls (R&D IC006N and IC006P) for 30 minutes on ice. Cells were then fixed using 4% PFA and permeabilized, followed by staining with primary antibodies anti-vimentin (Abcam ab24525; 1:200) and anti-aSMA (Abcam ab5694; 1:400) or their respective isotype controls for 30 minutes. For KI67 experiments, KI67 (Abcam AB16667) was used at 1:300. Anti-chicken-AF488 (for vimentin; Jacksons 703-545-155) and anti-rabbit-AF568 (for SMA or KI67) (Invitrogen A10042) secondary antibodies were used at 1:100. The data was recorded using BD FACS ARIA Fusion, ARIA III or Thermo Attune Nxt flow cytometer. The data were analysed on BD FACS Diva and FCS Express 7.0.

An unstained sample was used to set the voltages. Single-stained samples, including CD26-PE, SCA1-AF700, AF488 secondary, AF568 secondary, vimentin primary-AF488 secondary, SMA or KI67 primary-AF568 secondary, and respective conjugated-isotype controls with secondary antibodies, were then recorded. Quadrant gates in PE/AF488, PE/AF568, PE/AF700, AF488/AF568, AF488/AF700, and AF568/AF700 plots were used to perform compensation. Compensation was achieved by ensuring that the X and Y medians in the lower-left quadrant of unstained and single-stained samples matched the X and Y medians in the upper-left and lower-right quadrants of single-stained tubes. The gates were readjusted to minimize background signal based on samples containing cells with only the respective isotype control and secondary antibodies. Data were then recorded from co-stained tubes of WT and *Snail Tg* dermal suspensions (co-stained with CD26-PE, SCA1-AF700, vimentin primary-AF488 secondary, and SMA or KI67 primary-AF568 secondary).

**Transwell migration assay**

NBDF, CD26$^+$, SCA1$^+$, or shRNA-transduced fibroblasts (50,000–100,000) were added to the upper chambers of 8-µm Transwell inserts in 10% FBS-containing DMEM, with the same media added to the bottom chamber, and incubated at 37 °C for 2–4 hours to allow cell attachment. After incubation, transwell inserts were washed with PBS, and fresh serum-free media was added to the top chamber. Transwell chambers were then placed in fresh wells containing serum-free media with either control buffer or Mindin. For inhibitor assays, serum-free media with either DMSO, PP2 (10 µM) (Millipore 529573), KB SRC 4 (10 µM) (R&D 1008345), or iCRT5 (50 µM) (gift from Dr. Ramanuj Dasgupta, A*STAR, Singapore) was added to the top chamber, 5 mins before placing the wells in Mindin-containing serum-free media. After 24 hours, the experiment was terminated, and transwells were stained with crystal violet (CV) solution (0.5% CV, 10% ethanol, 4% PFA in PBS) and imaged after cleaning the top chamber. Fold change in migration was calculated as: (Number of cells/field migrated in the treated well)/(Number of cells/field in a migrated in control well)

**Cell localisation analysis**

The algorithm for calculating the spatial probability distribution is described on GitHub (https://github.com/skinlab-sunnyk/celllocalization). Briefly, the images were rotated to align the epidermis parallel to the horizontal axis. The X and Y coordinates (in pixel) were derived by marking the nuclei in the epidermal compartment and nuclei positive for either SCA1 or CD26 in the dermal compartment, using the Fiji Image J multipoint tool (78). These

coordinates were entered into the algorithm, which calculated the distance of a given cell in the dermis from its nearest cell in the epidermis and assigned a bin number based on this distance. The empirical probability of finding a cell as a function of distance from the epidermis was calculated as:

P (cell type, bin number) = number of cells in a bin/total number of cells counted.

Bin size = 5 μm was used based on the average size of the nucleus in the dermis. Welch's t-test was used to compare means of corresponding bins between WT and *Snail Tg*, *Snail Tg* and *Snail Tg/Min KO*, or WT and *Snail Tg/Min KO* mice. To visualise how the likelihood of finding a cell relative to the expected niche, the spatial probability P was scaled with its maximum value (P/Pmax). P/Pmax was used to generate the heatmap, where the width and intensity were maximum when P/Pmax = 1. The code for this analysis can be found at https://github.com/skinlab-sunnyk/celllocalization.

**Nearest neighbour analysis**

The algorithm for calculating the spatial nearest neighbour distances w.r.t. distance from the epidermis is shared on GitHub (https://github.com/skinlab-sunnyk/nnanalysis). Briefly, the images were rotated to align the epidermis parallel to the horizontal axis. The X and Y coordinates (in pixels) were derived by marking the nuclei in the epidermal compartment and nuclei positive for CD26 in the dermal compartment using Fiji Image J multipoint tool (78). These coordinates were entered into the algorithm, which calculated the distance of a given cell in the dermis from its nearest cell in the epidermis and assigned a bin number based on this distance. The distance matrix was created with the distance of each $CD26^+$ cell with every other $CD26^+$ cell in a given section. The algorithm then extracts the distance to the nearest neighbour of each cell. The average distance to the nearest neighbour in a given bin for a given section is calculated as $(\sum D_i)/n$ where $D_i$ is the distance to the nearest neighbour of the ith cell in a given bin and n is the number of cells in that bin. Bin size = 5 μm was used based on the average size of the nucleus in the dermis. Welch's t-test was used to compare the corresponding bins between WT and *Snail Tg*, *Snail Tg* and *Snail Tg/Min KO*, or WT and *Snail Tg/Min KO* mice. The code for this analysis can be found at https://github.com/skinlab-sunnyk/nnanalysis.

**Colony formation**

For co-culture assays, $CD26^+$ and $SCA1^+$ fibroblasts were seeded in a 3.5 mm dish at low density (~10,000 cells). 24-hours post-seeding, cells were serum-starved overnight and then treated with either buffer or Mindin. 24-hours post-treatment, the media was removed, cells were washed with PBS, and 1000 undifferentiated keratinocytes were added per dish along with low-calcium E-media. 7-days post adding keratinocytes, the cells were fixed and stained with crystal violet for counting colonies. For conditioned media experiments, $CD26^+$ fibroblasts were seeded in a 12-well dish. At ~80%-90% confluency, cells were serum-starved and then treated with either buffer or Mindin for 24-hours. 24-hours post-treatment, the media was removed, and fresh low-calcium E-media was added. 48-hours later, the conditioned media was collected. Keratinocytes were seeded in a 24-well format (100 cells/well). 24-hours post-seeding, the media was changed with a 1:1 mixture of fresh low-calcium E-media and conditioned media. 7 days after adding the conditioned medium, the cells were fixed and stained with crystal violet for counting colonies.

The fold change was calculated as follows: colonies counted in treated/average colonies counted in control.

## Supplementary data

## Supplementary Figure S1:

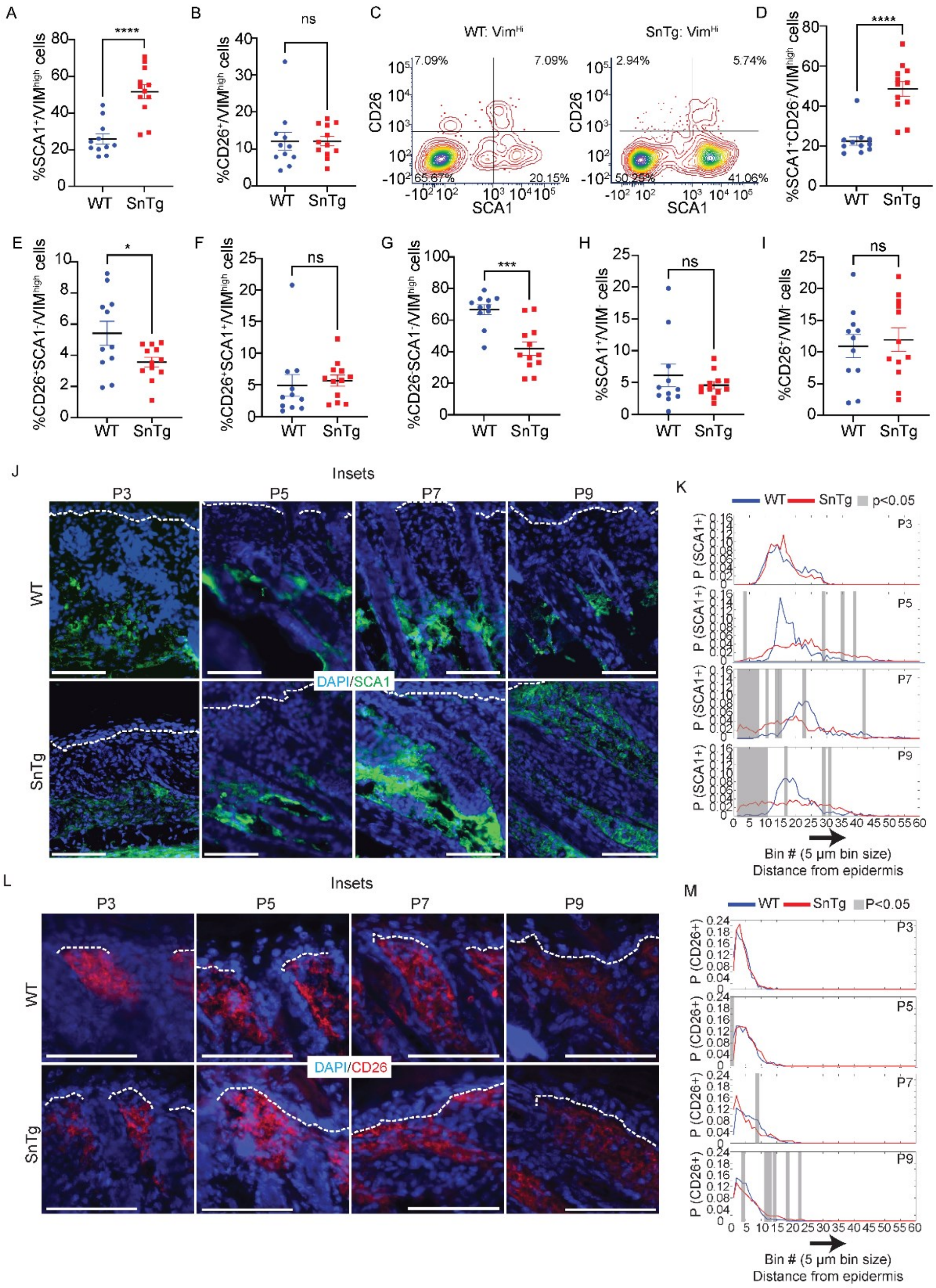

**Supplementary Figure S1: Proportion and Spatial distribution of SCA1$^+$ dermal fibroblasts are altered in *SnTg* skin.**

Flow cytometry data showing proportions of (A) SCA1$^+$/VIM$^{high}$ and (B) CD26$^+$/VIM$^{high}$ in Wildtype (WT) and *Snail transgenic (SnTg)* skin. (C) Representative plots showing VIM$^{High}$ cells gated for CD26 and SCA1in WT (left panel) and *SnTg* (right panel) skin. Proportion of (D) SCA1$^+$CD26$^-$/VIM$^{high}$, (E) CD26$^+$SCA1$^-$/VIM$^{high}$, (F) CD26$^+$SCA1$^+$/VIM$^{high}$, (G) CD26$^-$SCA1$^-$

/VIM$^{high}$, (H) SCA1$^+$/VIM$^-$, (I) CD26$^+$/VIM$^-$. Data shows mean±SEM and each dot represents an individual mouse; n=11 for WT and n=12 *SnTg*; p-values were calculated using Welch's t-test (A, B, D-I). (J) Insets for Figure 1E. Scale bar = 50 µm (K) Plots of the spatial probability distribution of SCA1$^+$ cells in WT and *SnTg* skin taken from P3 (n=3 WT and *Snail Tg*), P5 (n=2 WT and n=4 *Snail Tg*), P7 (n=3 WT and n=4 *Snail Tg*), and P9 (n=6 WT and n=8 *Snail Tg*) pups. The x-axis corresponds to successive 5 µm thick bins starting from the epidermis towards the dermis. (grey bars represent bins where p<0.05, using Welch's t-test). (L) Insets for Figure 1G. (M) Plots of the spatial probability distribution of CD26$^+$ cells in WT and *SnTg* skin taken from P3 (n=2 WT and n=3 Snail Tg), P5 (n=2 WT and n=3 *Snail Tg*), P7 (n=3 WT and n=3 *Snail Tg*), and P9 (n=4 WT and n=6 *Snail Tg*) pups. The x-axis corresponds to successive 5 µm thick bins starting from the epidermis towards the dermis. (grey bars represent bins where p<0.05, using Welch's t-test).

**Supplementary Figure S2:**

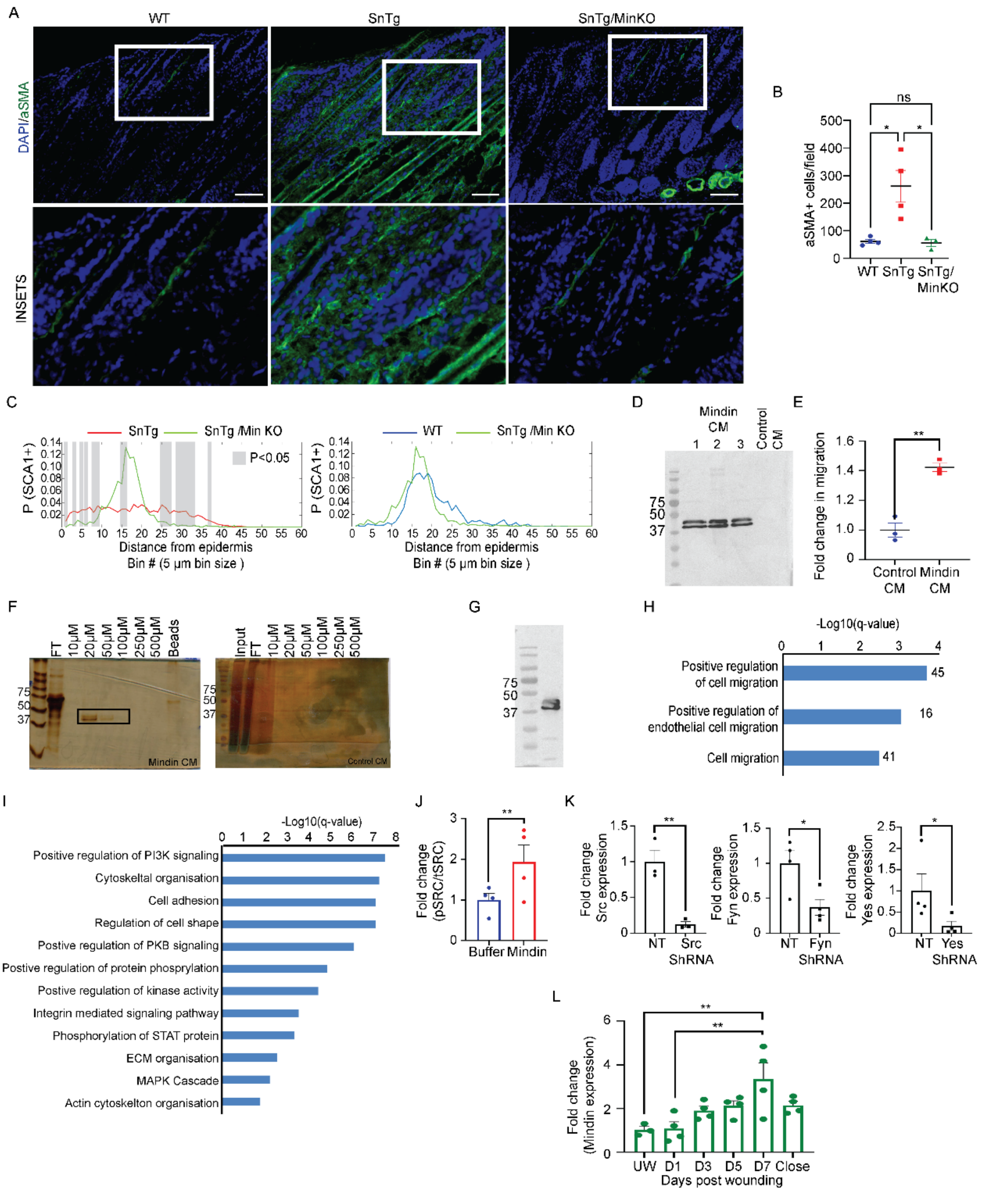


**Figure S2. Analysis of Mindin-induced migration in SCA1⁺ fibroblasts.** (A) Immunofluorescence of nuclei marked with DAPI (blue) and aSMA (green) in WT, *SnTg* and *SnTg/Min KO* skin. White boxes in the top panel mark the magnified region shown as insets in the bottom panel. (B) Quantification of aSMA⁺ dermal cells/field in WT (n=4), *SnTg* (n=4) and *SnTg/Min KO* (n=3) (each dot represents individual mice) (C) Comparison of the spatial probability distribution of SCA1⁺ cells between *SnTg* and *SnTg/Min KO* (Top panel) and WT and *SnTg/Min KO* (bottom panel). Each successive bin# on the x-axis corresponds to successive 5 µm steps below the epidermis. (n=6 for WT, n=8 for *SnTg* and n=4 for *SnTg/Min KO*; the area shaded in grey represents bins where p<0.05, calculated using Welch's t-test on corresponding bins). WT and *SnTg* are the same data as in Figure 1. (D) Western blot for Mindin using conditioned media (CM) from 3 batches of Mindin-producing

CHO cells (Mindin CM 1-3) and control CHO cells containing empty vector (Control CM). (E) Transwell assay to measure migration of fibroblasts in the presence of either Control CM or Mindin CM (n = 3 technical replicates) (F) Silver staining of gel using elutions from Ni-NTA purification of Mindin from conditioned media of histidine-tagged Mindin producing CHO cells showing lanes for Flow through (FT), elution with 10-500mM imidazole buffers, followed by beads after elution (left panel). Silver staining of conditioned media from control CHO cells with empty vector subjected to the same purification protocol (right panel). (G) Western blot for Mindin using purified recombinant Mindin post dialysis and concentration (H) Gene set enrichment analysis (GSEA) showing biological processes associated with migration, using the differentially upregulated genes from Mindin-treated fibroblasts. (I) GSEA of sub-list of Mindin upregulated genes associated with cell migration and positive regulation of cell migration. (J) Quantification of a western blot for pSRC/tSRC w.r.t. to buffer control (n=4). (K) qPCR for comparison of RNA expression for *Src*, *Fyn* and *Yes* kinases in the cells transduced with Non-targeting (NT) shRNA and *Src*, *Fyn* or *Yes* shRNA, respectively (n≥3). (L) qPCR for Mindin expression at 3-, 5-, 7- and 10-days post wounding (dpw) relative to unwounded skin (n≥3 mice for each group). Wounds closed on 10dpw. Data represent the mean±SEM. p-values were calculated by Ratio paired t-test (J, K), Welch's t-test (E),1-way ANOVA followed by Tukey's post hoc analysis (B and L) (*p < 0.05, **p < 0.01, ***p < 0.001, ****p < 0.0001 and ns (p>0.05) is non-significant).

## Supplementary Figure S3:

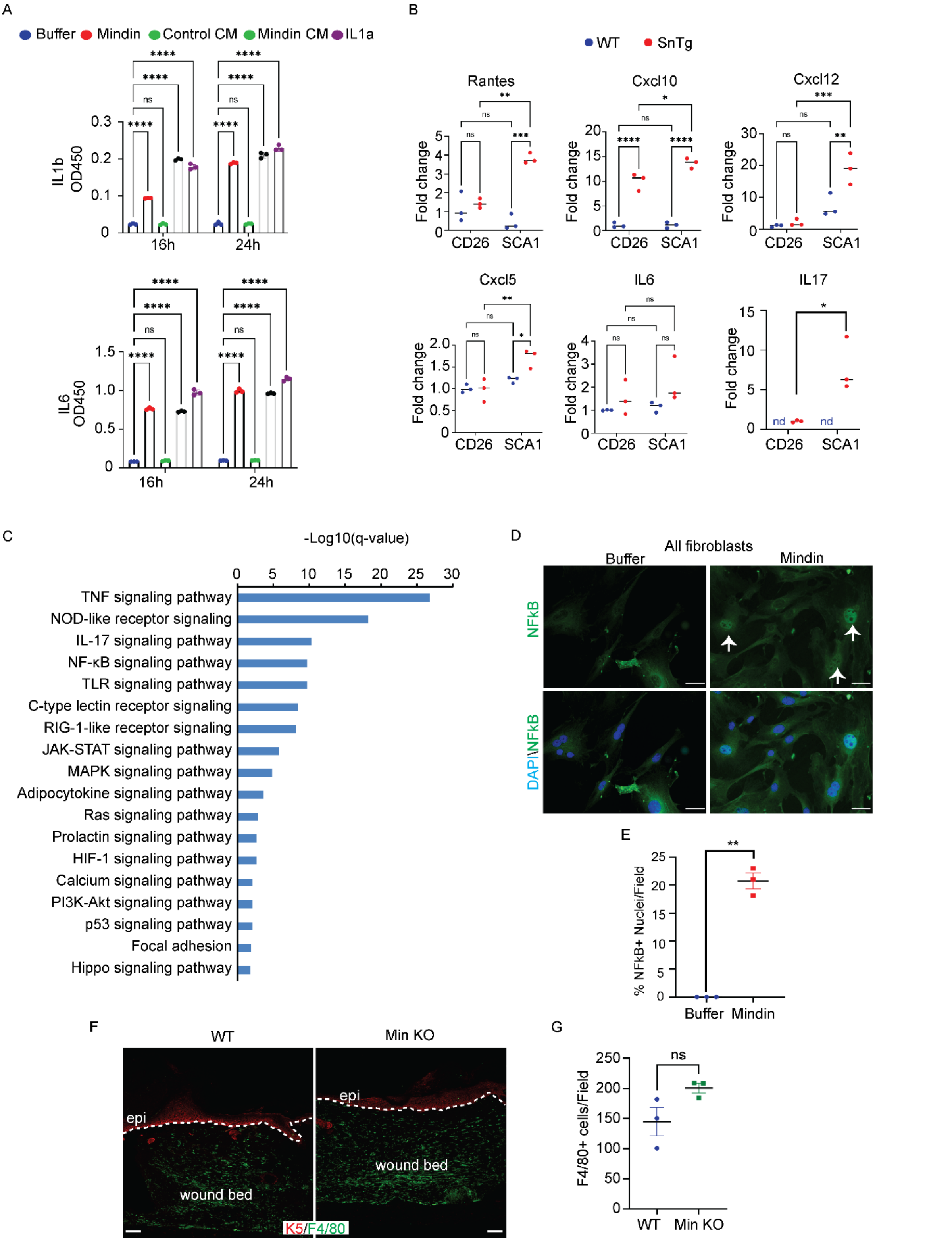


**Figure S3: Mindin activates inflammatory pathways in fibroblasts** (A) ELISA for IL1b (top panel) and IL6 (bottom panel) from fibroblasts treated with either buffer, Mindin, Control CM, Mindin CM or IL1a (positive control) (n=3; each dot represents an individual replicate) for 16 and 24 hours (B) qPCR for inflammatory cytokines expression in $CD26^{+}SCA1^{-}$ (CD26) and $SCA1^{+}CD26^{-}$ (SCA1) fibroblasts isolated from WT and *Snail Tg* mice skin. (n=3; each

dot represents individual mice; nd = not detected) (C) Enriched KEGG pathways terms based on GSEA of genes upregulated (1715 genes, FC>1.5, q<0.05) upon treatment of fibroblasts with Mindin. (D) IF staining for NFkB (green) and DAPI (blue) in unsorted mixed fibroblasts treated with either buffer or Mindin. White arrows point to a nuclear NFkB (scale bar = 50 µm). (E) Percentage of cells with NFkB+ nuclei/field (n=3). (F) IF staining for F4/80 and K5 in WT and *Min KO* skin sections post-wounded day 7 (scale bar = 100 µm) and (G) quantification of F4/80$^{+}$ cells/field in the wound bed (n=3; each dot represents data from individual mice). Data represents the mean ± SEM. p-values were calculated by Welch's t-test (E, G) and 2-way ANOVA followed by Tukey's post hoc analysis (A and B). *p<0.05, **p<0.01, ***p<0.001, ****p<0.0001 and ns (P>0.05) is non-significant.

**Supplementary Figure S4:**

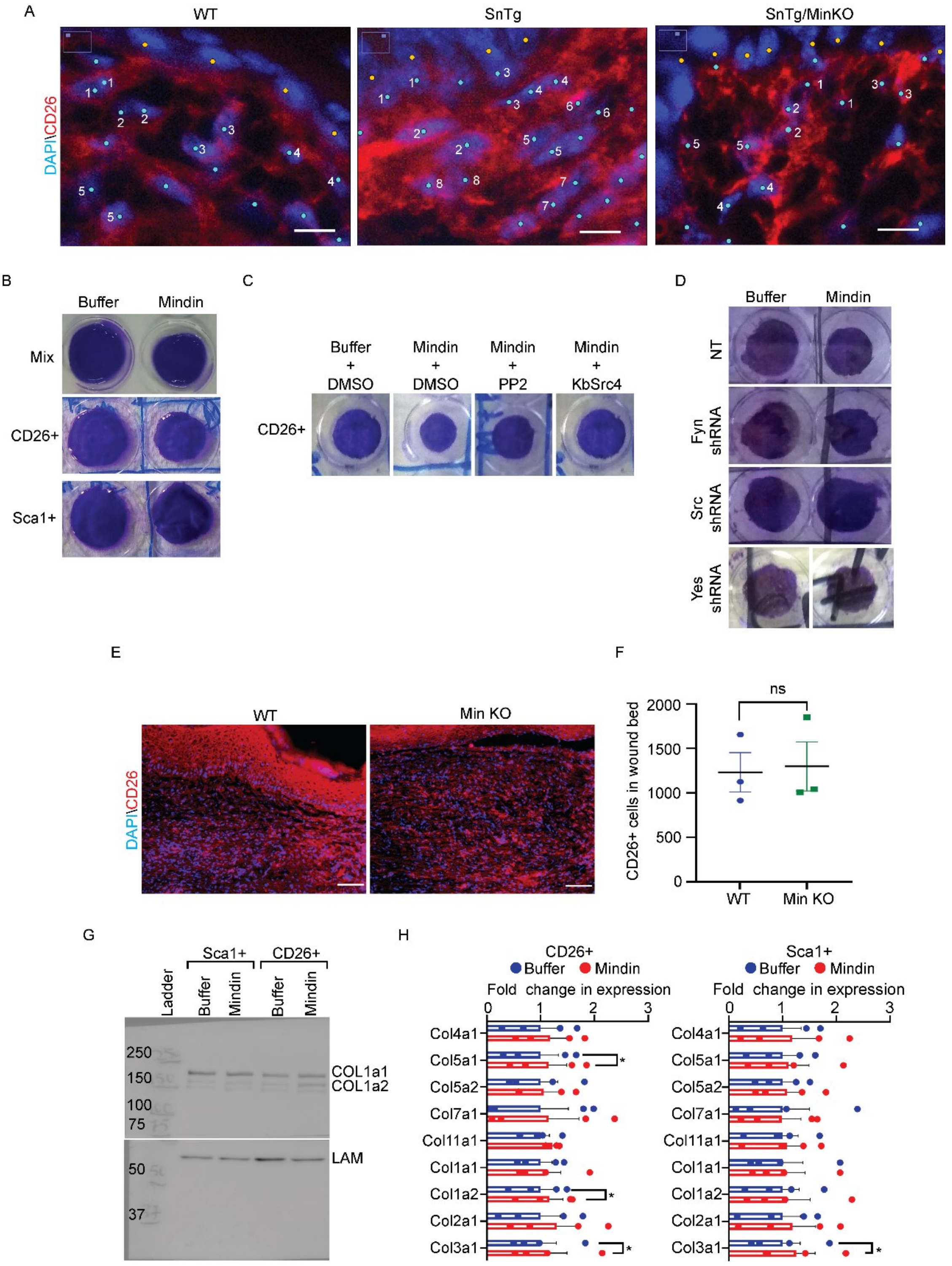


**Figure S4. CD26⁺ fibroblasts contract and produce collagen in response to Mindin.** (A) IF of CD26 (Red) and DAPI (blue) in WT, *SnTg*, and *SnTg/Min KO* skin sections (scale bar = 5 µm). Numbers refer to representative neighbouring cell pairs that are used to calculate intercellular distance between nearest neighbours. (B) Representative images for collagen gels seeded with either mixed fibroblasts, CD26+fibroblasts or SCA1+ fibroblasts treated

with either Buffer of Mindin, (C) Representative images of collagen gels seeded with CD26+ fibroblasts and treated with Buffer + DMSO, Mindin + DMSO, Mindin + PP2 or Mindin + KbSrc4. (D) Representative images of collagen gels seeded with CD26+ fibroblasts transduced with either Non-targeting (NT), *Src*, *Fyn*, or *Yes* shRNA and treated with either buffer or Mindin. (E) IF of CD26 (Red) and DAPI (blue) in WT and *Min KO* in wounded skin sections 7 days post-wounding (scale bar = 50 μm) and (F) its quantification. (G) Western blot showing bands for Collagen 1a1 (COL1a1) and Collagen 1a2 (COL1a2) and Lamin B1(LAM) in SCA1$^+$ and CD26$^+$ fibroblasts treated with either buffer control or Mindin. (H) RNA expression of different collagen subtypes quantified by RT-PCR in Mindin or buffer-treated CD26$^+$ and SCA1$^+$ fibroblasts (n = 4). Data represent the mean ± SEM. p-values were calculated by Welch's t-test (F), and ratio-paired t-test (H) (*p < 0.05 and ns (p>0.05) is non-significant).

**Supplementary Figure S5:**

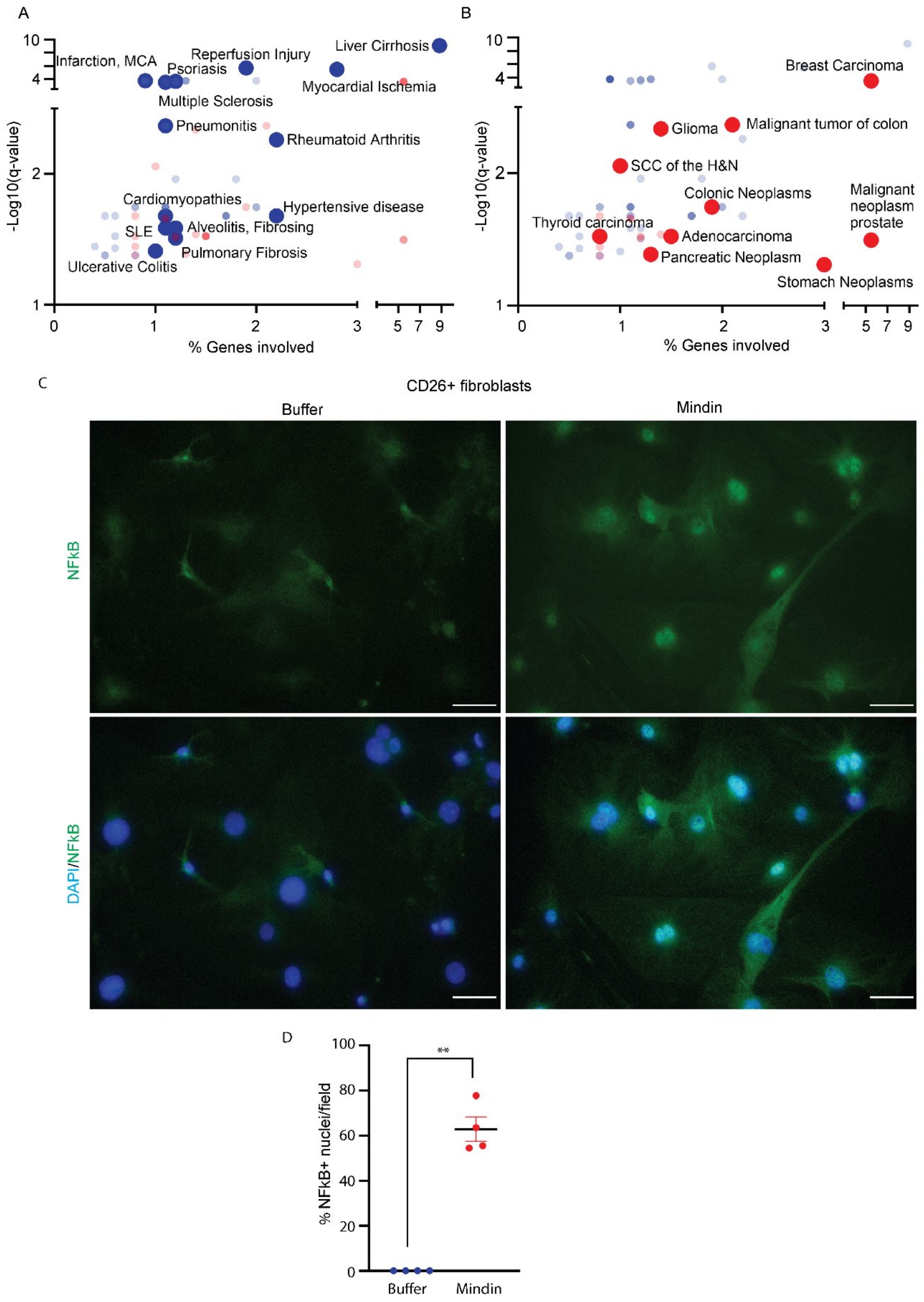


**Figure S5: Mindin induces CAF-like properties in fibroblasts** (A-B) GSEA of genes upregulated in Mindin-treated fibroblasts revealed terms associated with (A) fibrotic and inflammatory diseases and (B) cancers (using the DisGeNET database). (Plotted as % genes of total upregulated genes (1715) in the list (x-axis) and -Log10(q-value) (y-axis). (C)

IF staining for NF-kB (green) and DAPI (blue) in CD26$^+$ fibroblasts treated with either buffer (left) or Mindin (right) for 24-hours (D) Percentage of cells with NF-kB+ nuclei/field (n=4). Data represent the mean ± SEM. p-values were calculated by Welch's t-test, (**$p < 0.01$).

**Supplementary Figure S6:**

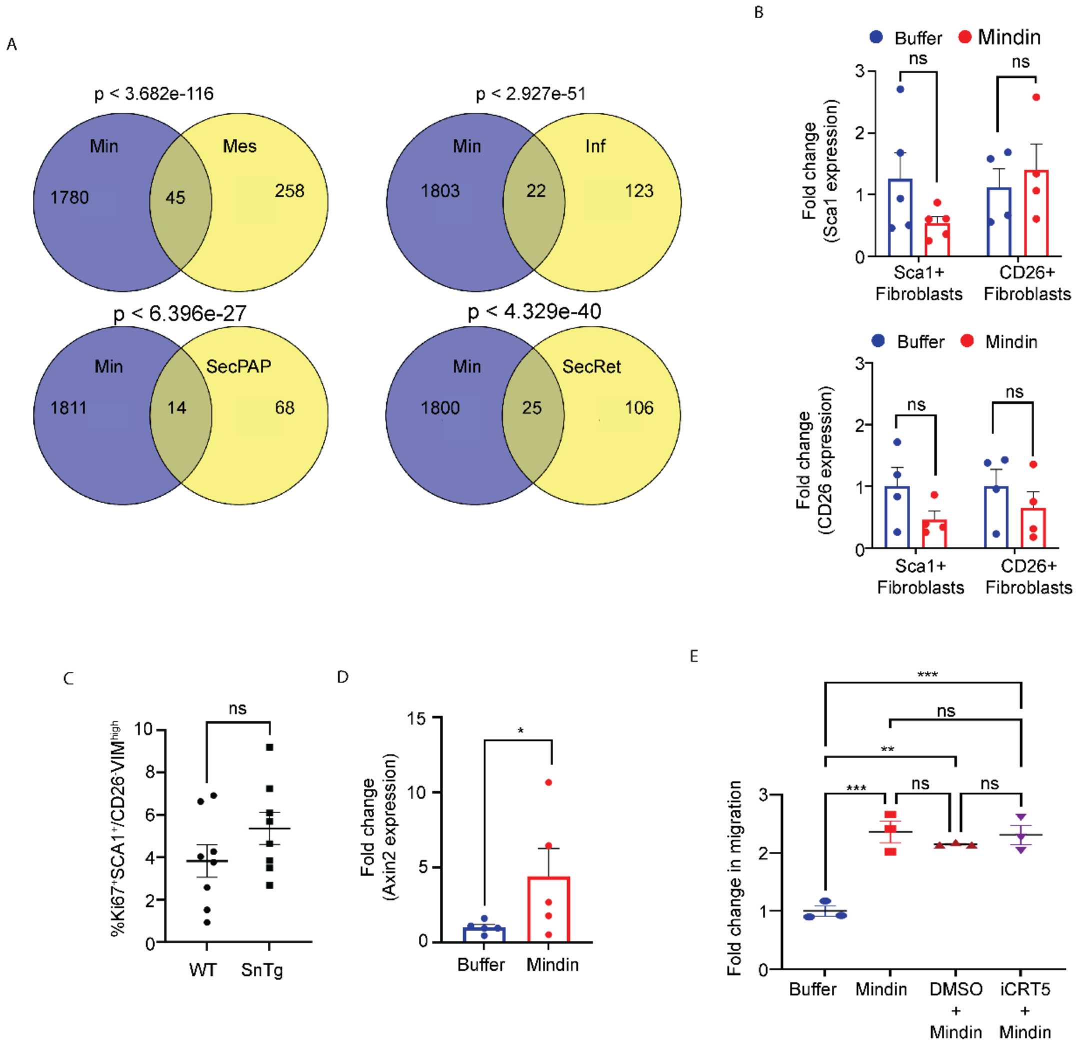


**Figure S6: SCA1+ fibroblasts are increased in *SnTg* skin.** (A) Venn-diagrams of overlapping genes upregulated in Mindin-treated human dermal fibroblasts and markers upregulated mesenchymal (mes), inflammatory (inf), secretory-papillary (SecPAP), secretory-reticular (SecRet) keloid fibroblasts. (B) Fold change in RNA expression of *Sca1* (left panel) and CD26 (right panel) in CD26$^{+}$ and SCA1$^{+}$ fibroblasts treated with either buffer of Mindin (n≥4). (C) %Ki67$^{+}$ of SCA1$^{+}$CD26$^{-}$VIM$^{+}$ cells in WT and *SnTg* skin (n=8 each dot represents individual mice). (D) Fold change in the RNA expression level of *Axin2* in SCA1$^{+}$ fibroblasts treated with either buffer of Mindin (n=5). (E) Migration of fibroblasts treated with either buffer, Mindin. Mindin+DMSO, or Mindin+iCRT5 (n=3). Data represent the mean ± SEM. p-values were calculated by hypergeometric test (A), 1-way ANOVA followed by Tukey's post hoc analysis (E), Welch's t-test (C), or ratio paired t-test (B, D). (*p < 0.05, **p < 0.01, ***p < 0.001 ns (p>0.05) is non-significant).

## Supplementary Table 1

| Parent Gate | Gate | WT1 | WT2 | WT3 | WT4 | WT5* | WT6* | SnTg 1 | SnTg 2 | SnTg 3 | SnTg 4 | SnTg5* | SnTg6* | Mean % WT | Mean % SnTg | p-Value |
|---|---|---|---|---|---|---|---|---|---|---|---|---|---|---|---|---|
| NA | All events | 10000 | 10000 | 10000 | 48526 | 10873 | 4714 | 10000 | 50000 | 50000 | 44403 | 19374 | 18634 | 100 | 100 | |
| All events | Cells | 8474 (84.74) | 8565 (85.65) | 8754 (87.54) | 46564 (95.96) | 8165 (75.09%) | 3010 (63.85%) | 8314 (83.14) | 41874 (83.75) | 43982 (87.96) | 41930 (94.43) | 13752 (70.98%) | 13907 (74.63%) | 82.14 | 82.48 | 0.953647 |
| Cells | Diag | 7003 (82.64) | 7753 (90.52) | 7692 (87.87) | 43592 (93.62) | 7078 (86.69%) | 2451 (81.43%) | 7416 (89.2) | 33854 (80.85) | 38120 (86.67) | 39021 (93.06) | 11455 (83.3%) | 12062 (86.73%) | 87.13 | 86.64 | 0.85212 |
| Diag | Live | - | - | - | - | 5685 (80.32%) | 1745 (71.2%) | - | - | - | - | 8759 (76.46%) | 9326 (77.32%) | 75.76 | 76.89 | 0.845465 |
| Diag/ Live | VIMHi | 949 (13.55) | 3395 (43.79) | 2005 (26.07) | 4391 (10.07) | 1136 (19.98%) | 268 (15.36%) | 2295 (30.95) | 3740 (11.05) | 4349 (11.41) | 13540 (34.7) | 1393 (15.9%) | 883 (9.47%) | 21.68 | 18.91 | 0.687514 |
| VIMHi | VhiSCA1- | 752 (79.24) | 2827 (83.27) | 1538 (76.71) | 2639 (60.1) | 667 (58.71%) | 180 (67.16%) | 953 (41.53) | 1622 (43.37) | 2172 (49.94) | 3745 (27.66) | 701 (50.32%) | 279 (31.6%) | 69.77 | 40.74 | 0.000999 |
| VIMHi | VhiCD26- | 837 (88.2) | 3222 (94.9) | 1872 (93.37) | 4515 (102.82) | 749 (65.93%) | 220 (82.09%) | 1871 (81.53) | 3021 (80.78) | 3572 (82.13) | 12987 (95.92) | 1230 (88.3%) | 757 (85.73%) | 86.01 | 85.73 | 0.95738 |
| **SMA±CD26±/SCA1-VIMHi** | | | | | | | | | | | | | | | | |
| VhiSCA1- | CD26+SMA- | 27 (3.59) | 29 (1.03) | 36 (2.34) | 276 (10.46) | 89 (13.34%) | 13 (7.22%) | 33 (3.46) | 108 (6.66) | 85 (3.91) | 102 (2.72) | 15 (2.14%) | 17 (6.09%) | 6.33 | 4.16 | 0.344544 |
| VhiSCA1- | CD26+SMA+ | 48 (6.38) | 70 (2.48) | 37 (2.41) | 154 (5.84) | 24 (3.6%) | 7 (3.89%) | 122 (12.8) | 167 (10.3) | 226 (10.41) | 215 (5.74) | 39 (5.56%) | 19 (6.81%) | 4.1 | 8.6 | 0.012391 |
| VhiSCA1- | CD26-SMA- | 280 (37.23) | 998 (35.3) | 736 (47.85) | 1345 (50.97) | 381 (57.12%) | 82 (45.56%) | 429 (45.02) | 948 (58.45) | 1110 (51.1) | 1953 (52.15) | 242 (34.52%) | 112 (40.14%) | 45.67 | 46.9 | 0.808121 |
| VhiSCA1- | CD26-SMA+ | 397 (52.79) | 1730 (61.2) | 729 (47.4) | 850 (32.21) | 172 (25.79%) | 77 (42.78%) | 369 (38.72) | 399 (24.6) | 750 (34.53) | 1454 (38.83) | 401 (57.2%) | 129 (46.24%) | 43.7 | 40.02 | 0.610284 |
| **SMA±SCA1±/CD26-VIMHi** | | | | | | | | | | | | | | | | |
| VhiCD26- | sca1+SMA- | 80 (9.56) | 194 (6.02) | 171 (9.13) | 276 (6.11) | 152 (20.29%) | 26 (11.82%) | 479 (25.6) | 1100 (36.41) | 747 (20.91) | 5286 (40.7) | 258 (20.98%) | 316 (41.74%) | 10.49 | 31.06 | 0.002014 |
| VhiCD26- | sca1+SMA+ | 65 (7.77) | 247 (7.67) | 185 (9.88) | 154 (3.41) | 50 (6.68%) | 35 (15.91%) | 513 (27.42) | 463 (15.33) | 835 (23.38) | 3249 (25.02) | 333 (27.07%) | 200 (26.42%) | 8.55 | 24.11 | 0.000109 |
| VhiCD26- | sca1-SMA- | 341 (40.74) | 1373 (42.61) | 978 (52.24) | 1345 (29.79) | 376 (50.2%) | 81 (36.82%) | 584 (31.21) | 1175 (38.89) | 1412 (39.53) | 3391 (26.11) | 239 (19.43%) | 112 (14.8%) | 42.07 | 28.33 | 0.028991 |
| VhiCD26- | sca1-SMA+ | 351 (41.94) | 1408 (43.7) | 538 (28.74) | 850 (18.83) | 170 (22.7%) | 76 (34.55%) | 295 (15.77) | 283 (9.37) | 577 (16.15) | 1061 (8.17) | 396 (32.2%) | 127 (16.78%) | 31.74 | 16.41 | 0.018238 |

**Supplementary Table 1:** Cell counts (% of parent gate) in WT and *SnTg* animals. p-value is calculated for % gated using Welch's t-test. * Live cells were gated for VIMHigh WT5, WT6, SnTg5 and SnTg6

**Supplementary Table 2:** Differentially expressed genes in Mindin-treated fibroblasts compared to buffer-treated fibroblasts (available as a separate Excel file).

**Supplementary Table 3:** GSEA for GOTERMS using upregulated genes from Mindin-treated fibroblasts (available as a separate Excel file).

**Supplementary Table 4:** KEGG pathway terms showing significant enrichment using upregulated genes from Mindin-treated fibroblasts (available as a separate Excel file).

## Supplementary Table 5:

| | WT1 | WT2 | WT3 | SnTg 1 | SnTg2 | SnTg3 | Mean WT | Mean SnTg | p-value |
|---|---|---|---|---|---|---|---|---|---|
| % CD45$^-$CD31$^-$ cells/All gated cells | 96.54 | 96.79 | 91.02 | 95.9 | 95.64 | 96.27 | 94.78 | 95.94 | 0.60314 |
| % CD45$^+$CD31$^+$ cells/All gated cells | 3.05 | 2.82 | 6.99 | 4.06 | 4.33 | 3.72 | 4.29 | 4.04 | 0.87104 |
| % SCA1$^+$CD45$^+$CD31$^+$/all gated cells | 0.57 | 0.65 | 0.79 | 0.72 | 0.96 | 0.9 | 0.67 | 0.86 | 0.12739 |
| % SCA1$^+$CD45$^-$CD31$^-$/all gated cells | 2.01 | 2.36 | 1.53 | 3.80 | 4.62 | 4.67 | 1.97 | 4.37 | 0.00315 |
| Ratio (SCA1$^+$CD45$^-$CD31$^-$: SCA1$^+$CD45$^+$CD31$^+$) | 3.54 | 3.63 | 1.93 | 5.3 | 4.82 | 5.2 | 3.03 | 5.11 | 0.05630 |

**Supplementary Table 5:** CD45$^-$CD31$^-$ SCA1$^+$ cells dominate over CD45$^+$CD31$^+$SCA1$^+$ cells.

**Supplementary Table 6: List of primers used**

| Gene | Forward primer | Reverse Primer |
|---|---|---|
| *Gapdh* | AGGTCGGTGTGAACGGATTTG | TGTAGACCATGTAGTTGAGGTCA |
| β-Actin (*Actb*) | GGGCTATGCTCTCCCTCAC | GATGTCACGCACGATTTCC |
| *Col1a1* | GCCAAGAAGACATCCCTGAAG | TCATTGCATTGCACGTCATC |
| *Col1a2* | TGCTGCTTGCAGTAACGTCG | TCAACACCATCTCTGCCTCG |
| *Col2a1* | GGGAATGTCCTCTGCGATGAC | GAAGGGGATCTCGGGGTTG |
| *Col3a1* | CTGTAACATGGAAACTGGGGAAA | CCATAGCTGAACTGAAAACCACC |
| *Col4a1* | CCTGGCACAAAAGGGACGA | ACGTGGCCGAGAATTTCACC |
| *Col5a1* | GCCCTCAGGGGTAACGAAAAC | GACTCGGTAGGCAACATCCG |
| *Col5a2* | TTGGAAACCTTCTCCATGTCAGA | TCCCCAGTGGGTGTTATAGGA |
| *Col7a1* | ACCACGTTTCTGACCGTGTC | AGCTGTGTCCACTAAATCTTGG |
| *Col11a1* | CCAGCGGGTCTTATGGGTC | TGGTAACATCAGCATGGTTCC |
| *Rantes* | CCTCACCATCATCCTCACTGCA | TCTTCTCTGGGTTGGCACACAC |
| *IL6* | CGTGGAAATGAGAAAAGAGTTGTG | CCAGTTTGGTAGCATCCATCATTTCT |
| *Cxcl10* | CCAAGTGCTGCCGTCATTTTC | GGCTCGCAGGGATGATTTCAA |
| *Cxcl5* | TGCGTTGTGTTTGCTTAACCG | CTTCCACCGTAGGGCACTG |
| *Cxcl3* | CTGCACCCAGACAGAAGTCAT | CCGTTGGGATGGATCGCTTT |
| *IL17* | GGAGAGCTTCATCTGTGTCTCTG | TTGGCCTCAGTGTTTGGACA |
| *Sca1* | AGGAGGCAGCAGTTATTGTGG | CGTTGACCTTAGTACCCAGGA |
| CD26 | ACCGTGGAAGGTTCTTCTGG | CACAAAGAGTAGGACTTGACCC |
| *Axin2* | TGACTCTCCTTCCAGATCCCA | TGCCCACACTAGGCTGACA |
| *Cxcl12* | TGCATCAGTGACGGTAAACCA | TTCTTCAGCCGTGCAACAATC |
| *aSma* | ATCGTCCACCGCAAATGC | AAGGAACTGGAGGCGCTG |
| *Tagln* | ACCAAAAACGATGGAAACTACCG | GTGAAGTCCCTCTTATGCTCCT |
| *Mcam* | AAGCTGGTCACTTTAACCACC | ATTCACTCTTACGAGTCGGGG |
| *Myh11* | AAGCTGCGGCTAGAGGTCA | CCCTCCCTTTGATGGCTGAG |
| *Myl6* | ATGGGTGCTGAAATCCGTCAT | CCGCCACTAGCATCTCTACTTC |
| *Sema3c* | GTGGACCACATTCTTAAAGGCA | GTCCTTGGATTGTCAGTTTCCAG |
| *Antxr1* | CAGAGAGGGAGGCTAACCGAT | GCAATCCGAGCCAACTGAGT |
| *Itga11* | TGCCCCAATGGAAACCAATG | CACTCGTGCGACCAGAGAG |
