# Supplementary material for: Mindin regulates fibroblast subpopulations through distinct Src family kinases during fibrogenesis
Source: JCI Insight. 2024 Dec 31;10(3):e173071. doi: 10.1172/jci.insight.173071 (PMC11948575; doi:10.1172/jci.insight.173071)

Full unedited blot/gel for Figure S4G

Ladder Blot Overlay

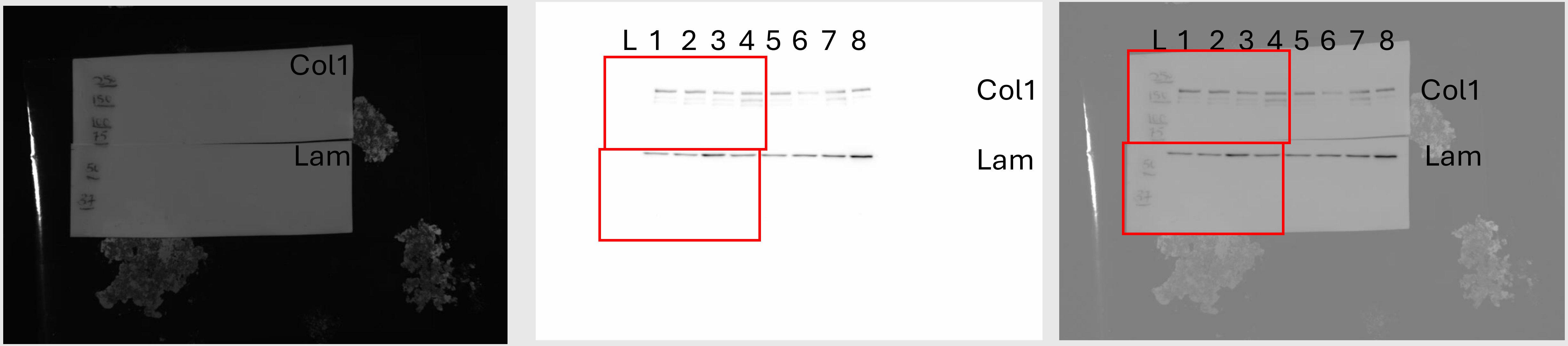

Lane	Sample
L	Ladder
1	Sca1 Buffer (Set 1)
2	Sca1 Mindin (Set 1)
3	CD26 Buffer (Set 1)
4	CD26 Mindin (Set 1)
5	CD26 Mindin (Set 2)
6	CD26 Buffer (Set 2)
7	Sca1 Mindin (Set 2)
8	Sca1 Buffer (Set 2)

Note: The region in the red box (lanes L, 1-4) was cropped and used for Figure S4E

Full unedited blot/gel for Figure 2D (top panel)

Ladder

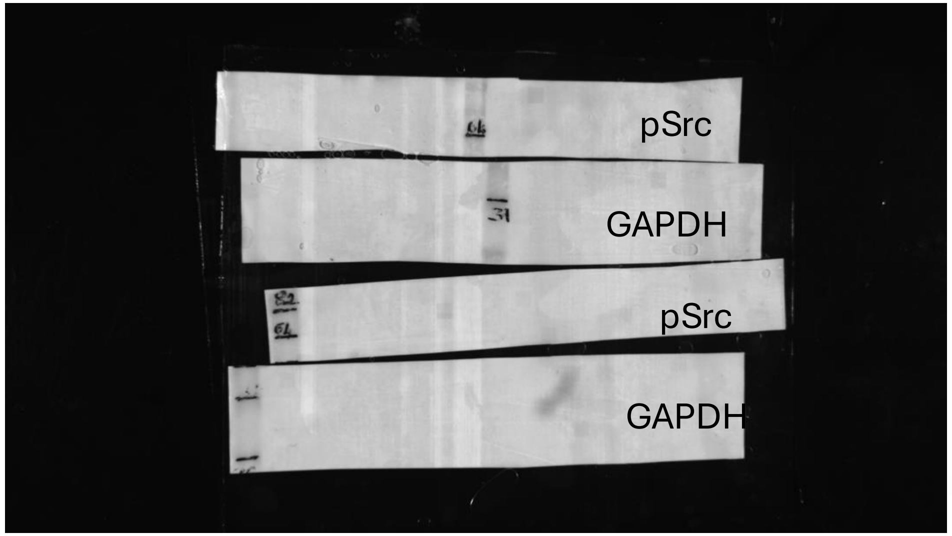

Blot

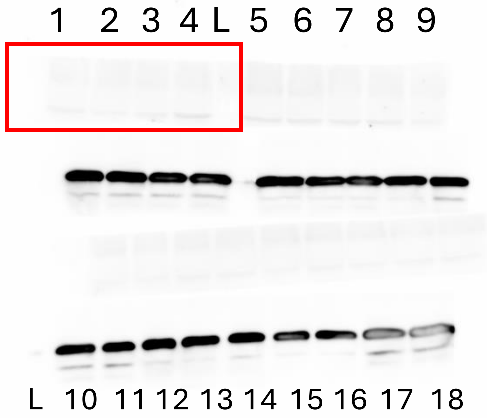

Overlay

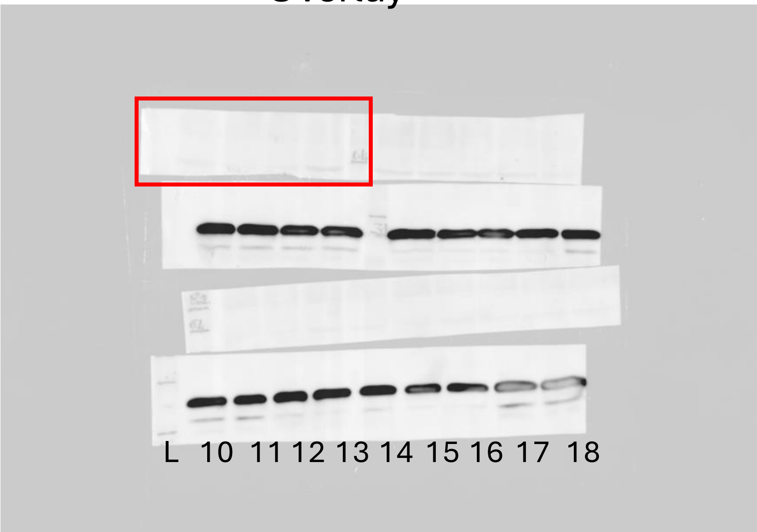

Lane	Sample
1	Untreated -1 (set 1)
2	Untreated -2 (set 1)
3	Buffer 15mins (set 1)
4	Mindin 15mins (set 1)
L	Ladder
5	Mindin (8 hrs) (set 2)
6	Mindin (12 hrs) (set 2)
7	Mindin (24 hrs) (set 2)
8	PDGF (24 hrs) (set 2)
9	Buffer (24 hrs) (set 2)

Lane	Sample
L	Ladder
10	Untreated (Set 3)
3	Buffer 15mins (set 3)
4	Mindin 15mins-1 (set 3)
L	Mindin 15 mins-2 (Set 3)
5	Buffer 15 mins (set 4)
6	Mindin 15 mins (set 4)
7	PDGF 15 mins (set 4)
8	Mindin 1 hr (set 5)
9	Buffer (1 hrs) (set 5)

Note: The region in red box containing lane 1-4, L was cropped, Contrast enhanced and used for Figure 2D (Top panel)

Full unedited blot/gel for Figure 2D (Bottom panel)

Ladder

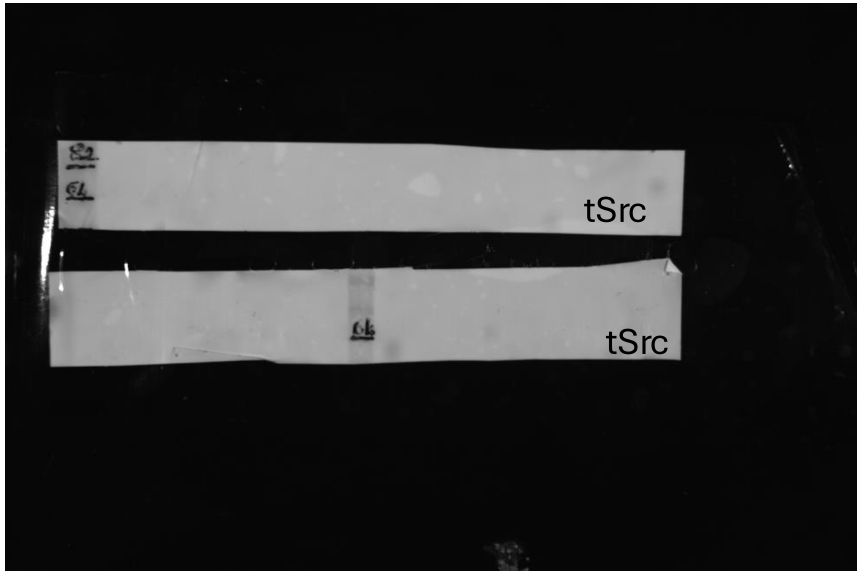

Blot

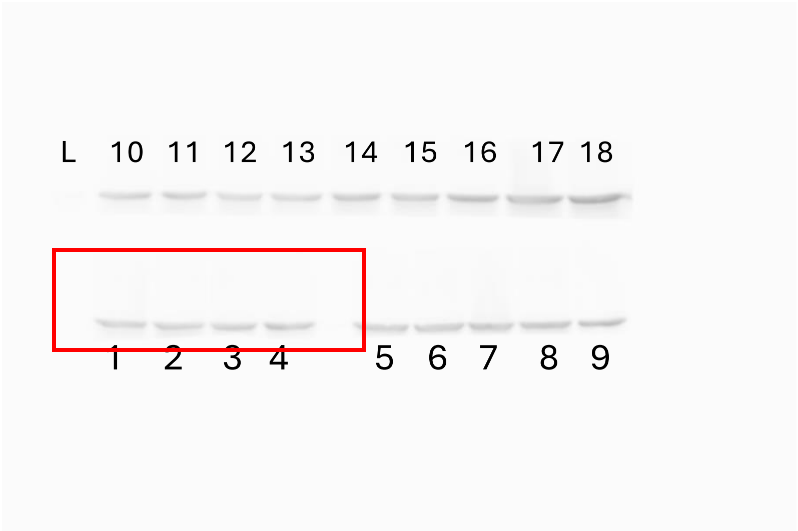

Overlay

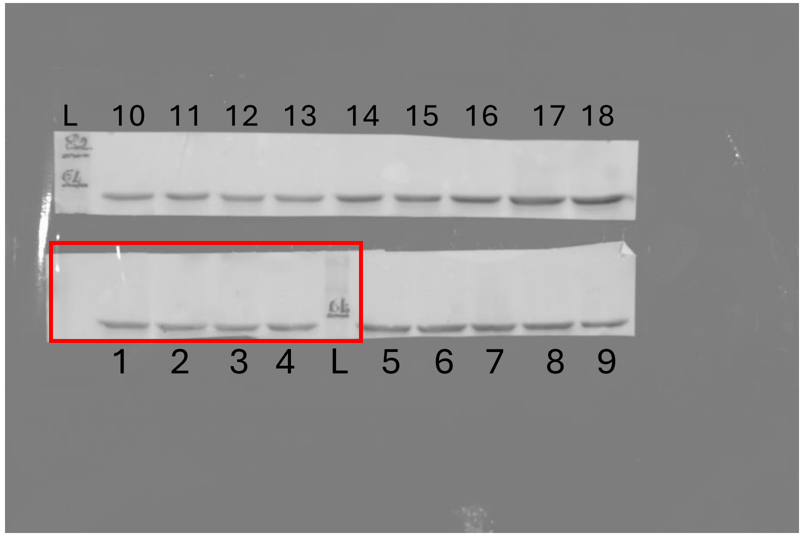

Lane	Sample
1	Untreated -1 (set 1)
2	Untreated -2 (set 1)
3	Buffer 15mins (set 1)
4	Mindin 15mins (set 1)
L	Ladder
5	Mindin (8 hrs) (set 2)
6	Mindin (12 hrs) (set 2)
7	Mindin (24 hrs) (set 2)
8	PDGF (24 hrs) (set 2)
9	Buffer (24 hrs) (set 2)

Lane	Sample
L	Ladder
10	Untreated (Set 3)
3	Buffer 15mins (set 3)
4	Mindin 15mins-1 (set 3)
L	Mindin 15 mins-2 (Set 3)
5	Buffer 15 mins (set 4)
6	Mindin 15 mins (set 4)
7	PDGF 15 mins (set 4)
8	Mindin 1 hr (set 5)
9	Buffer (1 hrs) (set 5)

Note: The region in red box containing lane 1-4, L was cropped and used for Figure 2D (Top panel)

Full unedited blot/gel for Figure S2D

Ladder

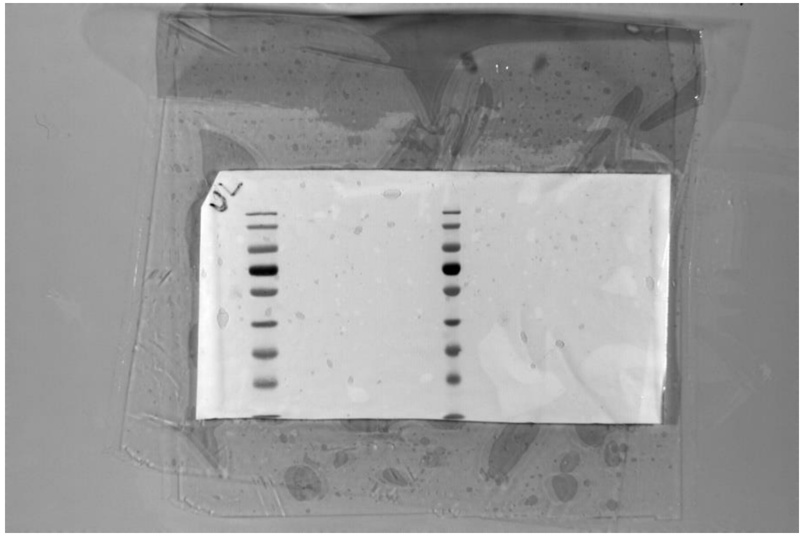

Blot

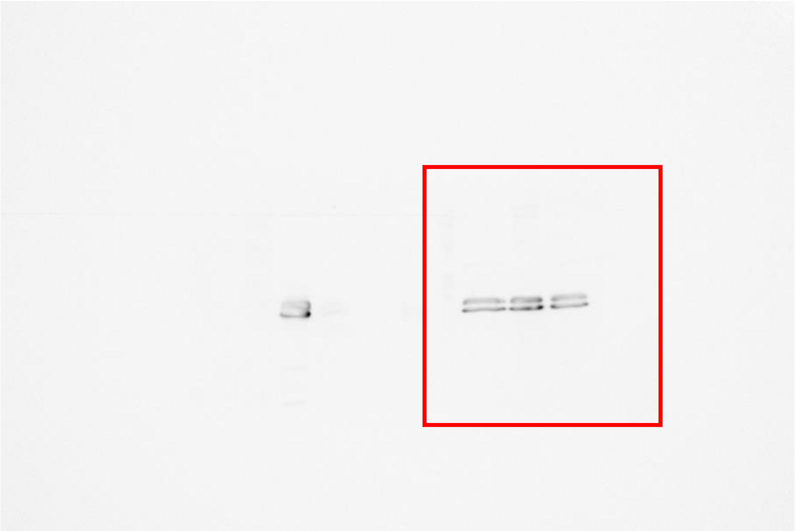

Overlay

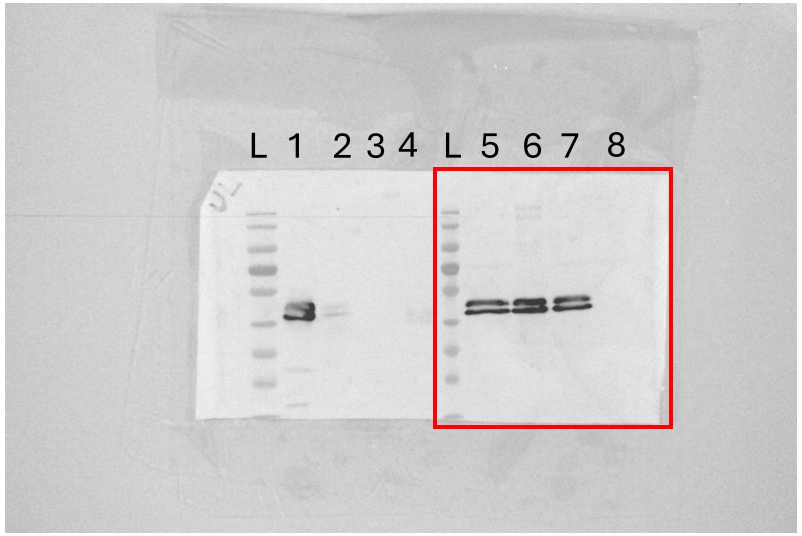

Lane	Sample
L	Ladder
1	Concentrated purified Mindin
2	Dialysed purified Mindin
3	Control elution
4	Pre dialysis purified Mindin
L	Ladder
5	Midin CM – ProCHO AT - 1
6	Midin CM – ProCHO AT – 2
7	Midin CM – ProCHO AT – 3
8	Control CM - ProCHO AT

Note: The region in red box containing lanes - L,5,6,7,8 was cropped and used for Figure S2D

Full unedited blot/gel for Figure S2G

Ladder

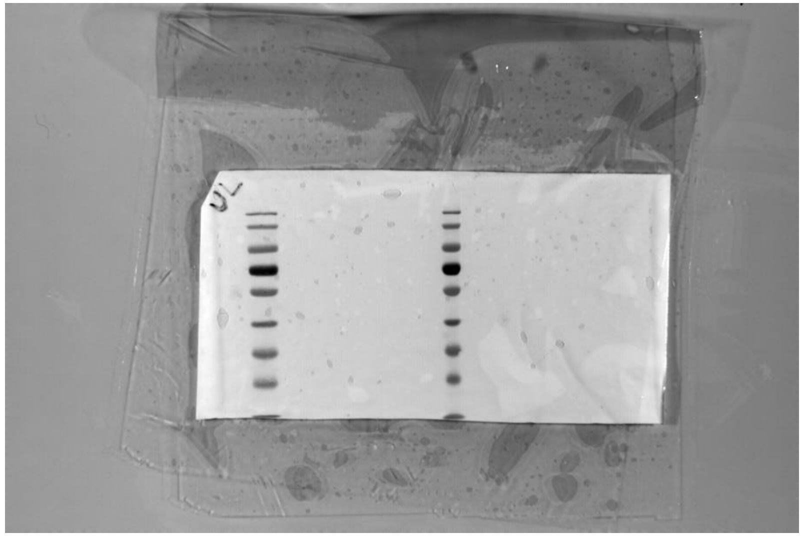

Blot

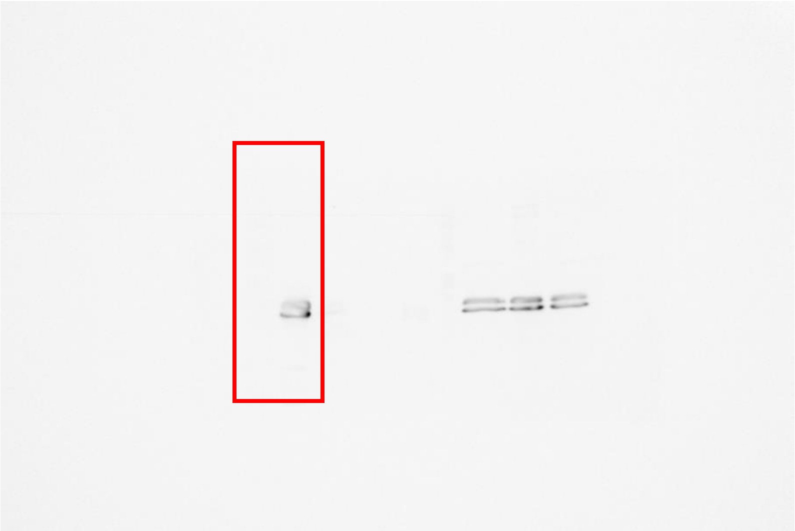

Overlay

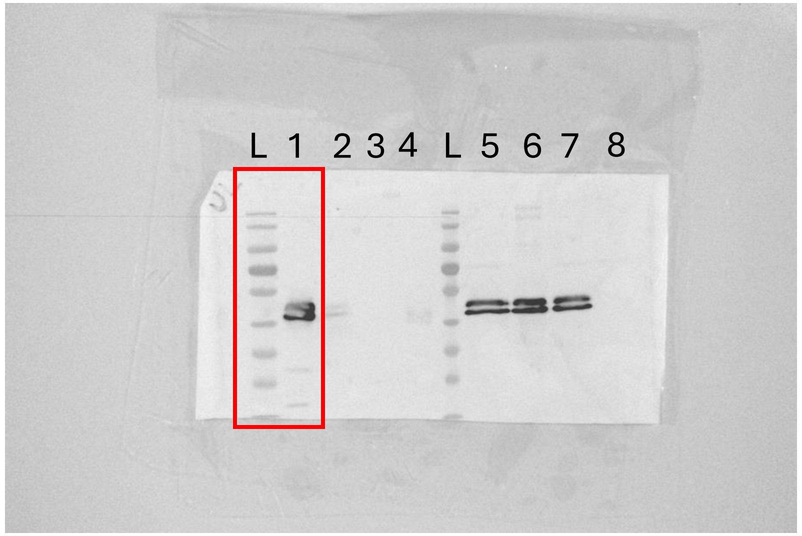

Lane	Sample
L	Ladder
1	Concentrated purified Mindin
2	Dialysed purified Mindin
3	Control elution
4	Pre dialysis purified Mindin
L	Ladder
5	Midin CM – ProCHO AT - 1
6	Midin CM – ProCHO AT – 2
7	Midin CM – ProCHO AT – 3
8	Control CM - ProCHO AT

Note: The region in red box containing lanes - L,1 was cropped and used for Figure S2G

Full unedited blot/gel for Figure S2F (left panel)

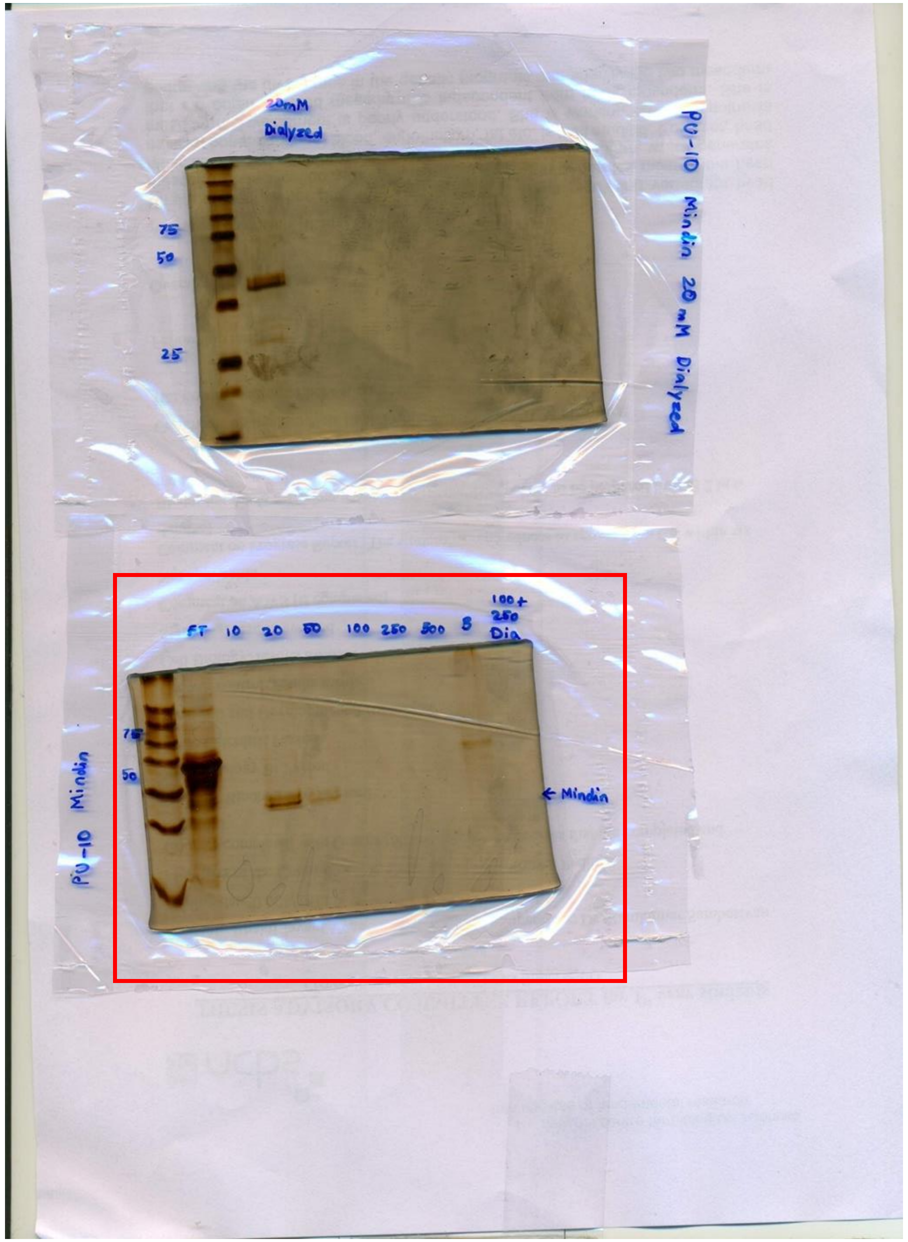

Note: The gel in red box containing lanes was cropped and used for Figure S2F

Full unedited blot/gel for Figure S2F (right panel)

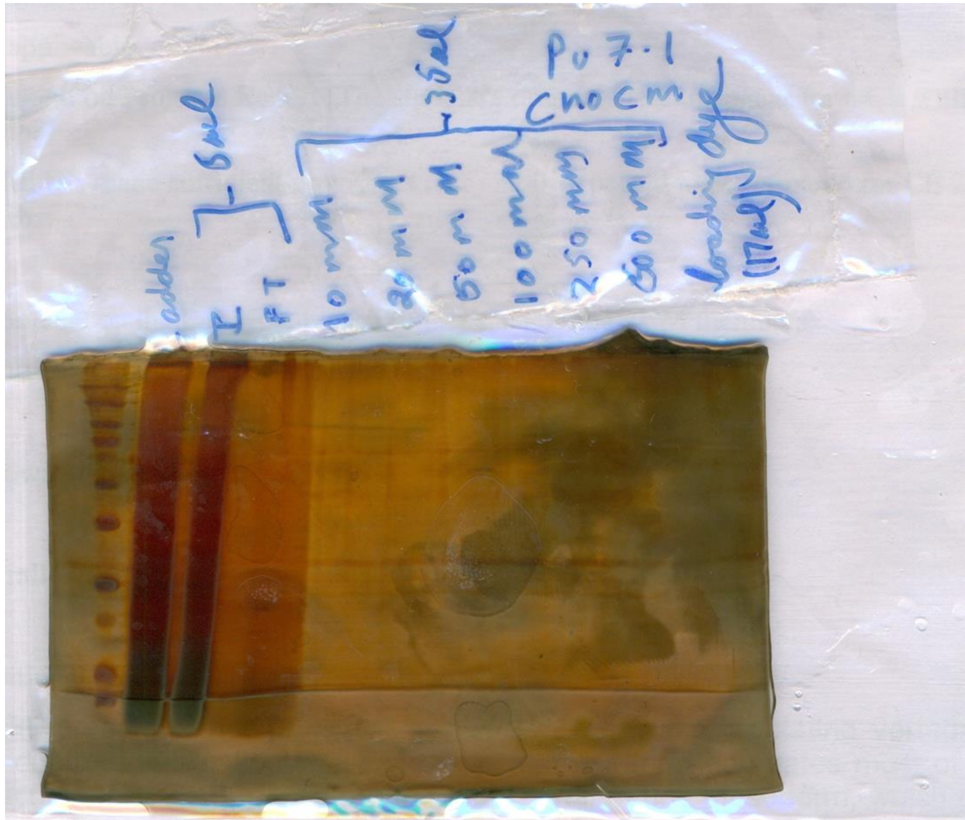

Supplement: Unedited blot and gel images [file jciinsight-10-173071-s145.pdf]
